# Menstruation and social inequities in Spain: a cross-sectional online survey-based study

**DOI:** 10.1186/s12939-023-01904-8

**Published:** 2023-05-17

**Authors:** Laura Medina-Perucha, Tomàs López-Jiménez, Constanza Jacques-Aviñó, Anna Sofie Holst, Carme Valls-Llobet, Jordina Munrós-Feliu, Cristina Martínez-Bueno, Diana Pinzón-Sanabria, Mª Mercedes Vicente-Hernández, Anna Berenguera

**Affiliations:** 1 Fundació Institut Universitari Per a La Recerca a L’Atenció Primària de Salut Jordi Gol I Gurina (IDIAPJGol), Gran Via de Les Corts Catalanes 587 Attic, 08007 Barcelona, Spain; 2 Universitat Autònoma de Barcelona, Cerdanyola del Vallès, Spain; 3 Universitat Pompeu Fabra, Barcelona, Spain; 4 Centro de Análisis Y Programas Sanitarios (CAPS), Barcelona, Spain; 5 Atenció a La Salut Sexual I Reproductiva (ASSIR) Muntanya/La Mina, Institut Català de La Salut, Barcelona, Spain; 6 Sexual and Reproductive Health Care Research Group (GRASSIR), Barcelona, Spain; 7 Servei d’Atenció a La Salut Sexual I Reproductiva (ASSIR). Direcció Assistencial d’Atenció Primària. Institut Català de La Salut, Barcelona, Spain; 8 University of Barcelona, Barcelona, Spain; 9 SomiArte Taller, Barcelona, Spain; 10 Departament d’Infermeria, Universitat de Girona, Girona, Spain

**Keywords:** Menstrual inequity, Period poverty, Menstrual health, Menstrual hygiene management, Menstruation, Women’s health, Social inequities, Androcentrism, Inequidad menstrual, Pobreza menstrual, Salud menstrual, Manejo menstrual, Menstruación, Salud de las mujeres, Inequidades sociales, Androcentrismo

## Abstract

**Background:**

Available research suggests that menstrual inequity has an impact on (menstrual) health outcomes and emotional wellbeing. It is also a significant barrier to achieve social and gender equity and compromises human rights and social justice. The aim of this study was to describe menstrual inequities and their associations with sociodemographic factors, among women and people who menstruate (PWM) aged 18–55 in Spain.

**Methods:**

A cross-sectional survey-based study was conducted in Spain between March and July 2021. Descriptive statistical analyses and multivariate logistic regression models were performed.

**Results:**

A total of 22,823 women and PWM were included in the analyses (Mean age = 33.2, SD = 8.7). Over half of the participants had accessed healthcare services for menstruation (61.9%). The odds for accessing menstrual-related services were significantly higher among participants with university education (aOR: 1.48, 95% CI, 1.13–1.95). Also, 57.8% reported having had partial or no menstrual education pre-menarche, with odds being higher among participants born in non-European or Latin American countries (aOR: 0.58, 95% CI, 0.36–0.93). Lifetime self-reported menstrual poverty was between 22.2–39.9%. Main risk factors for menstrual poverty were identifying as non-binary (aOR: 1.67, 95% CI, 1.32–2.11), being born in non-European or Latin American countries (aOR: 2.74, 95% CI, 1.77–4.24), and not having a permit to reside in Spain (aOR: 4.27, 95% CI, 1.94–9.38). Completed university education (aOR: 0.61, 95% CI, 0.44–0.84) and no financial hardship < 12 months (aOR: 0.06, 95% CI, 0.06–0.07) were protective factors for menstrual poverty. Besides, 75.2% reported having overused menstrual products due to lack of access to adequate menstrual management facilities. Menstrual-related discrimination was reported by 44.5% of the participants. Non-binary participants (aOR: 1.88, 95% CI, 1.52–2.33) and those who did not have a permit to reside in Spain (aOR: 2.11, 95% CI, 1.10-4.03) had higher odds of reporting menstrual-related discrimination. Work and education absenteeism were reported by 20.3% and 62.7% of the participants, respectively.

**Conclusions:**

Our study suggests that menstrual inequities affect a high number of women and PWM in Spain, especially those more socioeconomically deprived, vulnerabilised migrant populations and non-binary and trans menstruators. Findings from this study can be valuable to inform future research and menstrual inequity policies.

**Supplementary Information:**

The online version contains supplementary material available at 10.1186/s12939-023-01904-8.

## Resumen

**Introducción:**

Investigación previa disponible indica que la inequidad menstrual tiene un impacto en los resultados de salud (menstrual) y en el bienestar emocional. Es también una barrera para la equidad social y de género. El objetivo de este estudio es evaluar la inequidad menstrual y las asociaciones con factores sociodemográficos, en mujeres y personas que menstrúan entre 18-55 años en España.

**Métodos:**

Este es un estudio transversal, basado en una encuesta, llevado a cabo en España entre marzo y julio de 2021. Se realizaron análisis descriptivos y modelos de regresión logística multivariados.

**Resultados:**

Los análisis se realizaron con los datos de 22,823 mujeres y personas que menstrúan. Más de la mitad de las participantes habían accedido a servicios sanitarios para la menstruación (60.5%). La probabilidad de acceder a servicios sanitarios para la menstruación fue significativamente más alta en participantes con educación universitaria (aOR: 1.48, 95% CI, 1.13-1.95). El 57.8% informó no haber tenido educación menstrual o que ésta fuera parcial, pre-menarquia; la probabilidad fue más alta en participantes que no habían nacido en países europeos o latinoamericanos (aOR: 0.58, 95% CI, 0.36-0.93). La pobreza menstrual durante el ciclo vital se reportó en el 22.2-39.9% de las participantes. Los principales factores de riesgo fueron identificarse como persona no binaria (aOR: 1.67, 95% CI, 1.32-2.11), nacer en países fuera de Europa o Latinoamérica (aOR: 2.74, 95% CI, 1.77-4.24), y no tener papeles para residir en España (aOR: 4.27, 95% CI, 1.94-9.38). Tener estudios universitarios (aOR: 0.61, 95% CI, 0.44-0.84) y no haber reportado problemas económicos en los últimos 12 meses (aOR: 0.06, 95% CI, 0.06-0.07) fueron factores protectores para la pobreza menstrual. Además, el 74.6% indicó haber sobreutilizado productos menstruales por no haber tenido acceso a espacios adecuados para el manejo menstrual. El 42.6% de las participantes comunicaron experiencias de discriminación menstrual. Participantes no binarios (aOR: 1.88, 95% CI, 1.52-2.33) y aquellas que no tenían papeles (aOR: 2.11, 95% CI, 1.10-4.03) presentaron una mayor probabilidad de indicar discriminación menstrual. El absentismo laboral y escolar fue indicado por el 18.3% y el 56.6% de las participantes respectivamente.

**Conclusiones:**

Nuestro estudio sugiere que la inequidad menstrual afecta a un número significativo de mujeres y personas que menstrúan en España y, especialmente, a aquellas en situaciones de mayor deprivación socioeconómica, algunos colectivos vulnerabilizados de personas migradas, y a personas no binarias y trans que menstrúan. Los resultados de este estudio pueden ser útiles para investigación futura, así como para el desarrollo de políticas públicas de equidad menstrual.

## Background

There is a growing attention towards the need to ensure menstrual health among women and people who menstruate (PWM) worldwide [1–4]. This is a significant shift from the deeply ingrained neglect and invisibilisation of menstruation and the menstrual cycle in social, political, economic and research spheres [5–7]. While there is an exponential increase of published research on menstrual health and menstrual hygiene management, research on menstrual inequity is still soundly scarce.

This study was framed based on a definition of *menstrual inequity* developed by the authors, based on data from the “Equity and Menstrual Health in Spain” project [8]. We understand menstrual inequity as* “the systematic and avoidable differences in the access to menstrual healthcare, education and knowledge, products, services and facilities for menstrual management, menstrual-related experiences of stigma and discrimination, and social, community, political and economic participation based on having a menstrual cycle and menstruating”*. We suggest that menstrual inequity is an umbrella term that encompasses period poverty [9, 10], and menstrual hygiene management [11]. It also collates with definitions of menstrual health [12] as it refers to the social factors and conditions that may have an impact on menstrual and other health outcomes. Throughout this publication, we will use the terms “menstrual poverty” instead of “period poverty” as “period” can be understood as an euphemism for “menstruation” [13]. The term “menstrual management” will be used, instead of “menstrual hygiene management”, as referring to “hygiene” could contribute to the prevailing menstrual taboo and stigma [14].

While evidence is limited, previous research has already suggested that menstrual inequities are associated with worse (menstrual) health outcomes, including the risk of bacterial infections [15]; barriers to diagnose menstruation-related health conditions (e.g., endometriosis) [6, 16–19]; a negative impact on emotional health and wellbeing [9, 20–22]; menstrual taboo, stigma and discrimination [13, 23]; absenteeism and presenteeism from schools, universities and workplaces, and subsequently reduced productivity [24–26]; misinformation associated with worse health outcomes [27, 28]; barriers for social, community and economic participation [8, 9, 23, 29]. Menstrual inequity also poses a barrier to achieve the Sustainable Development Goals [4]. Moreover, available frameworks [12] and recent literature reviews [30, 31] suggest the access to education, healthcare, products, and facilities (e.g., including water, sanitation and hygiene), as well as stigma, discrimination, and community participation as main social inequities of menstrual health.

Available evidence has not focused on menstrual inequity as a comprehensive concept, but rather on disaggregated forms of menstrual inequity (e.g., menstrual poverty). Research on menstrual management and menstrual poverty has also largely focused on communities from the Global South and young people [2]. While research initiatives are starting to be echoed in the Global North [8, 20, 21, 32–34], it is urgent to assess menstrual inequity comprehensively in our context. Doing so could remarkably contribute to make menstrual inequities socially and politically visible, and challenge the assumptions on menstrual inequity not being an issue in high-income countries.

One of the steps to understand menstrual inequities is to assess how these social inequities may differ by population groups. This could contribute to understand how social inequities influence, not only menstrual, but other health outcomes associated with menstrual access and management. Very importantly, it could inform policymaking to ensure menstrual justice and social equity. The Spanish government has recently approved new menstrual leaves and a decrease in the taxes applied to menstrual products (from 10 to 4%, as it corresponds to essential goods). Therefore, at a time when menstrual policies are being debated and approved in Spain, it is timely that we can ensure and support evidence-based policymaking. In this article, we aimed at responding to the following questions: “How many women and PWM are estimated to be affected by menstrual inequities in Spain? What groups of women and PWM might be particularly at risk for these social inequities in Spain?”. The aim of this study was to describe menstrual inequities and their associations with sociodemographic factors, among women and PWM aged 18–55 in Spain. This research is part of a larger mixed-methods study, the “Equity and Menstrual Health in Spain” project. To our knowledge, this is the first study to explore menstrual inequities comprehensively in Spain.

## Methods

A cross-sectional study using an online survey was conducted among a non-nationally representative sample of women and PWM living in Spain between 24th of March and 8th of July 2021. Inclusion criteria were being 18–55 years old, having menstruated at least once in the last year, being able to understand and provide informed consent, and understanding Spanish to complete the questionnaire. Main exclusion criteria included having entered menopause. STROBE guidelines for reporting cross-sectional studies have been followed.

### Sampling, recruitment and data collection

A minimum of 1,535 women and PWM participants were required. The sample size, calculated for the “Equity and Menstrual Health in Spain” project, was based on power calculations considering a “menstrual inequity” proxy. Sample size for the study was based on available evidence on menstrual hygiene management, given the lack of published research on menstrual inequity. Maximum indetermination of the main variable (proportion of 50%) was assumed. These assumptions were in order to obtain a precision of 2.5% in the confidence intervals. These estimates have been calculated assuming an alfa risk of 5%. PASS software was used for the sample size calculations [PASS 15 Power Analysis and Sample Size Software (2017). NCSS, LLC. Kaysville, Utah, USA].

Sampling was non-probabilistic and purposive. Several recruitment strategies were used, including social media platforms (e.g., Twitter), primary healthcare centres, sexual and reproductive healthcare centres, non-governmental and other local organisations. Snowballing techniques were widely used. Data were collected using an online questionnaire that included questions on menstrual health, menstrual inequity and sociodemographic factors. The questionnaire was designed by the research team, a group of interdisciplinary experts including psychologists, medical doctors, public health professionals and midwives, among others. Several meetings were organised to devise the questionnaire, as guided by previous research and guidelines on questionnaire design [35]. The questionnaire was piloted, took around 20 minutes to be completed, and was available in Spanish. The current publication will only present menstrual inequity-related data.

The LimeSurvey platform was used for online data collection and management. LimeSurvey is considered a secure, web-based software designed to securely conduct online surveys (https://www.limesurvey.org). Even though most data were collected online, researchers also collected data face-to-face to ensure the participation of hard-to-reach populations and those who may not have access to online platforms. This was done at sexual and reproductive health centres, a service for sex workers, and a food bank.

### Variables

Conceptualisations of menstrual inequity changed throughout the study, based on the team’s experience conducting the study and most recent evidence. For this reason, variables’ operationalisation was modified from the projects’ original protocol. Variables were operationalised for data analysis as follows.

Sociodemographic variables included: age, gender and trans identification, country of birth, administrative situation, employment situation, completed education, and financial problems in the last 12 months.

Based on the concept of menstrual inequity developed by the research team [8], menstrual inequities were assessed based on the following variables: 1) previous access to menstrual health services [a) overall; b) for symptoms associated with the use of menstrual products]; 2) no menstruation and menstrual cycle knowledge pre-menarche; 3) wanting more information on menstruation and the menstrual cycle; 4) normalisation of dysmenorrhea [based on a) considering not necessary to seek professional assistance when feeling menstrual pain during the menstrual cycle; and b) believing severe dysmenorrhea is “normal”]; 5) menstrual poverty [considering a) financial problems to buy menstrual products; and b) financial problems to afford preferred menstrual products]; 6) prolonged menstrual products’ use due to no products available; 7) prolonged menstrual products’ use due to no access to menstrual management facilities; 8) menstruation-related experiences of stigma and discrimination; 9) embarrassment to buy or ask for menstrual products; 10) fear of menstrual staining in public; 11) menstruation concealment; 12) embarrassment to talk about menstruation; 13) less able to pay attention or deal with day-to-day tasks during menstruation; 14) less productive during menstruation; 15) menstrual-related work absenteeism; 16) menstruation-related school absenteeism; and 17) declining social plans when menstruating.

### Data analysis

Descriptive statistics were performed for each variable to identify asymmetric distributions. Categorical variables were expressed as frequencies (percentage) and continuous variables as mean (SD) or median (interquartile range, IQR). We used the Chi-square test to assess differences between groups. Multivariate logistic regression models were constructed to evaluate associations between sociodemographic factors and menstrual inequity variables. Statistical significance was set at 0.05 and all tests were 2-tailed. Analyses were performed using SPSS 25.0 (SPSS Inc., Armonk, NY: IBM Corp), and Stata/MP 17.0 (StataCorp LLC, TX).

## Results

A total of 22,823 women and PWM participated in the study, most online and 78 face-to-face. Mean age was 33.2 (*SD* = 8.7), ranging between 18 and 55 years old. Most identified their gender as women (96.8%) and 3.2% as non-binary/other; 0.8% self-identified as trans. Most participants were born in Spain (93.4%) and held Spanish nationality (95.9%). Almost half were employed full-time (47.5%), 17.1% were studying full-time, 8% were receiving unemployment benefits or COVID-19 unemployment benefits, and 5% were unpaid homemakers or caregivers as their main occupation. More than half had completed university education (69.4%). 32.9% identified as caregivers for someone else (e.g., children). Almost half had had financial problems in the year preceding the study; 30.9% sometimes or a few times and 11.9% always or many times (see Table 1).

**Table 1:** Sociodemographic characteristics (*N* = 22,823)

Variable	N (22,823)	%
**Age (18–55)**	*M* = 33.2 (*SD* = 8.7)	
**Gender identification**		
Women	22,100	96.8%
Non-binary/Other	723	3.2%
**Trans**		
Yes	175	0.8%
Don’t know	155	0.7%
No	22,493	98.6%
**Place of birth**		
Spain	20,943	93.4%
Europe	841	3.8%
Latin America	501	2.2%
Other	126	0.6%
**Administrative situation**		
Spanish Nationality	21,815	95.9%
Permanent Residency	721	3.2%
Temporary Residency	174	0.8%
No permit/in process	49	0.2%
**Employment situation**		
Working full-time	10,834	47.5%
Working part-time	3,914	17.1%
Self-employed	2,050	9.0%
Studying full-time	3,896	17.1%
Studying part-time	1,934	8.5%
Unemployment /COVID-19 benefits	1,831	8.0%
Pension or retirement	163	0.7%
Unpaid carer/houseworker	1,134	5.0%
**Completed education**		
University education	15,811	69.4%
Secondary education	6,728	29.5%
Primary education	217	1.0%
No formal education completed	35	0.2%
**Caregiving for someone else**		
Yes	7,518	33.1%
No	15,183	66,9%
**Financial problems in the last year**		
Always/many times	2,707	11.9%
Sometimes/a few times	7,056	30.9%
Never	12,582	55.1%

Our study did not include a representative sample of women and PWM living in Spain. However, in order to support the contextualization of our findings, a comparative between our study’s data and Spanish National Statistics Institute (INE) representative data can be found in the Supplementary File 1. As s indicated in the supplementary material, there was a higher proportion of younger women and PWM in our study, compared to women living in Spain in 2020. Besides, the inclusion of migrant communities was low in our study (6.6.%), compared to the population living in Spain (23.6%). Our sample included a higher proportion of women and PWM with completed university education, and significantly less participants who were unemployed, retired or receiving pensions or other benefits. Data on self-reported financial hardship in the preceding 12 months could be compared with representative data on financial satisfaction. When comparing these data, our data does not significantly differ from the representative data available. This information should be considered when interpreting the findings presented in this article.

Main findings are reported under distinct sections: 1) access to healthcare services for menstrual health; 2) access to menstrual education and knowledge; 3) use of menstrual products, menstrual management and menstrual poverty; 4) taboo, embarrassment and experiences of stigma and discrimination; and 5) social and community participation. Stratified descriptive analyses on menstrual inequity by sociodemographic characteristics can be found in the Supplementary File 2.

### Access to healthcare services for menstrual health

While over half of the participants (61.9%) had accessed healthcare services for menstrual-related consultations, 15% did not think it was necessary to access healthcare services for menstruation and the menstrual cycle, and 22.3% had not accessed but wanted to access. Participants sought professional assistance from healthcare professionals (59.3%), alternative and complementary medicine professionals (7.7%), social care professionals (4.8%), and teachers (1.6%). Consultations’ satisfaction (*M* = 3.3, *SD* = 1.2) was variable (range of 1–5).

**Fig. 1:**
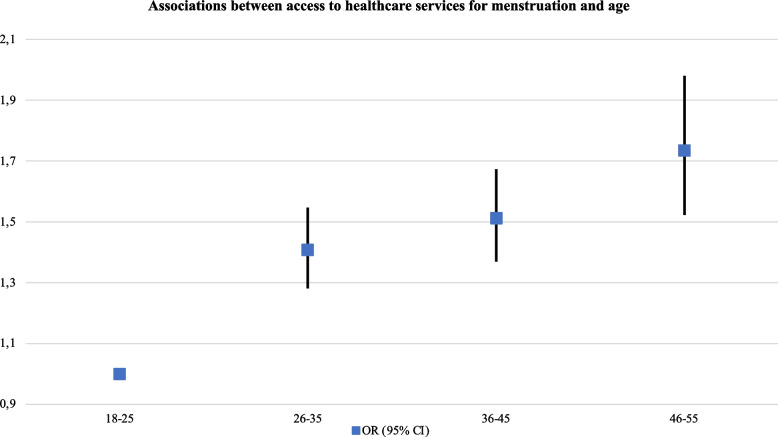
Associations between access to healthcare service for menstruation and age

**Fig. 2:**
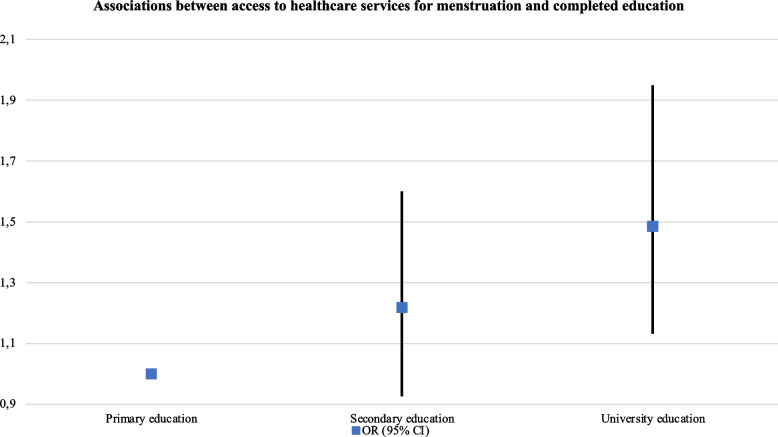
Associations between access to healthcare services for menstrual and completed education

The odds to access to healthcare services for menstruation significantly increased with age (e.g., for participants aged 46–55: aOR: 1.74, 95% CI, 1.52–1.98), among participants working full-time (aOR: 1.15, 95% CI, 1.04–1.28), those who were self-employed (aOR: 1.23, 95% CI, 1.09–1.39) or participants receiving a pension or retired (aOR: 1.41, 95% CI, 1.00–1.99). They were also significantly higher among participants who had completed university education (aOR: 1.48, 95% CI, 1.13–1.95). On the other hand, the odds significantly decreased in participants with a permanent Spanish residency (aOR: 0.69, 95% CI, 0.57–0.84), and among those who had never had financial problems in the last 12 months (aOR: 0.75, 95% CI, 0.68–0.82) (see Table 2 and Figs. 1 and 2.

**Table 2:** Associations between access to healthcare services for menstruation and sociodemographic characteristics (*N* = 21,418)

	**aOR (95%CI)**	***p*** ** value**
**Age**		
18–25	1.00	
26–35	1.41 (1.28–1.55)	< 0.001
36–45	1.51 (1.37–1.67)	< 0.001
46–55	1.74 (1.52–1.98)	< 0.001
**Gender**		
Women	1.00	
Non-binary/other	1.07 (0.87–1.30)	0.530
**Trans**		
No	1.00	
Yes	0.78 (0.54–1.13)	0.182
I don’t know	0.95 (0.64–1.40)	0.789
**Place of birth**		
Spain	1.00	
Latin America	0.89 (0.75–1.05)	0.159
Europe	1.01 (0.81–1.27)	0.912
Other	0.96 (0.65–1.42)	0.837
**Administrative situation**		
Spanish nationality	1.00	
Permanent residency	0.69 (0.57–0.84)	< 0.001
Temporal residency	0.77 (0.55–1.07)	0.120
No permit/in process	0.63 (0.35–1.13)	0.119
**Employment situation**		
Working full-time	1.15 (1.04–1.28)	0.006
Working part-time	1.11 (1.00–1.23)	0.054
Self-employed	1.23 (1.09–1.39)	0.001
Studying full-time	1.02 (0.90–1.14)	0.786
Studying part-time	1.10 (0.99–1.23)	0.073
Unemployment or COVID19 benefits	1.04 (0.92–1.18)	0.541
Pension or retirement	1.41 (1.00–1.99)	0.048
Unpaid carer/houseworker	1.07 (0.93–1.22)	0.352
**Completed education**		
Primary education	1.00	
Secondary education	1.22 (0.93–1.60)	0.160
University education	1.48 (1.13–1.95)	0.004
**Financial problems < 12 months**		
Always/Many times	1.00	
Some/A few times	0.93 (0.84–1.02)	0.128
Never	0.75 (0.68–0.82)	< 0.001

### Access to menstrual education and knowledge

Almost half (45.3%) reported having had partial information on menstruation and the menstrual cycle at menarche, and 12.5% did not know what menstruation was at that time. Around half (50.4%) did not feel prepared to menstruate at menarche. Most participants had initially learned about menstruation and the menstrual cycle through their families (71.4%), school (40.9%) or friends (37.6%). Menstrual education at the time of data collection was mostly through the internet (60%), health professionals (44.3%), friends (38.3%), social media networks (35.1%), magazines or books (19.7%), and family members (13.9%). Most participants (73.9%) reported  being interested in having more information on menstruation and the menstrual cycle, while 3.1% were not interested in this topic. In relation to participants’ views on dysmenorrhea, 13.2% believed that dysmenorrhea was “normal”, and 4% did not think seeking medical assistance for dysmenorrhea was necessary. On the contrary, 54.7% indicated that experiencing severe dysmenorrhea could be a sign of a health condition, and 47.6% believed that experiences of dysmenorrhea should be consulted with a health professional.

**Fig. 3:**
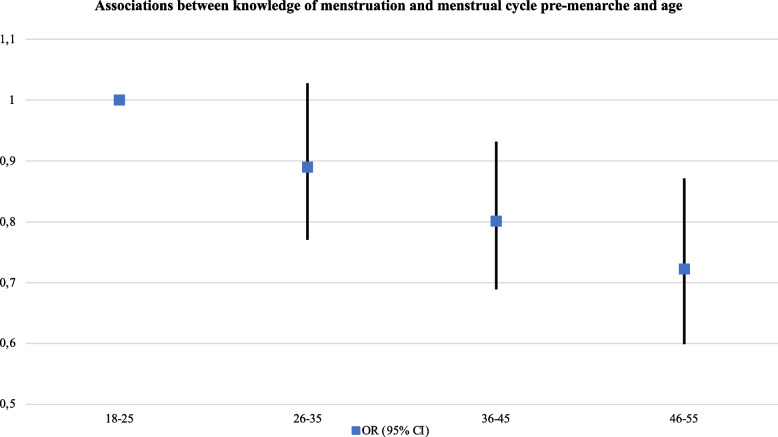
Associations between knowledge of menstruation and menstrual cycle pre-menarche and age

The odds for knowing what menstruation and the menstrual cycle were pre-menarche, were significantly reduced as age increased (ages 46–55: aOR: 0.72, 95% CI, 0.60–0.87), and among participants not born in Europe or Latin America (aOR: 0.58, 95% CI, 0.36–0.93). Participants working (aOR: 1.23, 95% CI, 1.06–1.43) or studying full-time (aOR: 1.22, 95% CI, 1.02–1.45), and participants with no financial problems in the 12 months preceding the study (aOR: 1.28, 95% CI, 1.12–1.46) had higher odds of knowing about menstruation and the menstrual cycle before menarche (see Table 3) (Fig. 3).

**Table 3:** Associations between access to menstrual education, knowledge and sociodemographic characteristics (*N* = 21,459)

	**Knowledge of menstruation and menstrual cycle pre-menarche (** ***N*** ** = 20,955)**		**Would like to have more information on menstruation and the menstrual cycle (** ***N*** ** = 20,199)**	
	**aOR (95%CI)**	***p*** ** value**	**aOR (95%CI)**	***p*** ** value**
**Age**				
18–25	1.00		1.00	
26–35	0.89 (0.77–1.03)	0.112	0.71 (0.62–0.82)	< 0.001
36–45	0.80 (0.69–0.93)	0.004	0.39 (0.34–0.45)	< 0.001
46–55	0.72 (0.60–0.87)	0.001	0.22 (0.19–0.26)	< 0.001
**Gender**				
Women	1.00		1.00	
Non-binary/other	1.26 (0.92–1.71)	0.147	0.92 (0.72–1.17)	0.475
**Trans**				
No	1.00		1.00	
Yes	0.69 (0.40–1.19)	0.182	0.36 (0.24–0.55)	< 0.001
I don’t know	0.79 (0.45–1.38)	0.405	0.78 (0.48–1.27)	0.319
**Place of birth**				
Spain	1.00		1.00	
Latin America	1.19 (0.92–1.52)	0.184	1.14 (0.92–1.41)	0.221
Europe	1.06 (0.76–1.48)	0.719	1.04 (0.79–1.38)	0.761
Other	0.58 (0.36–0.93)	0.024	0.80 (0.49–1.30)	0.359
**Administrative situation**				
Spanish nationality	1.00		1.00	
Permanent residency	0.91 (0.68–1.21)	0.518	0.88 (0.69–1.13)	0.329
Temporal residency	1.08 (0.65–1.82)	0.761	0.69 (0.45–1.06)	0.091
No permit/in process	0.53 (0.25–1.10)	0.088	2.86 (0.86–9.48)	0.086
**Employment situation**				
Working full-time	1.23 (1.06–1.43)	0.006	0.87 (0.76–0.98)	0.029
Working part-time	1.22 (1.05–1.42)	0.010	1.08 (0.94–1.21)	0.285
Self-employed	1.17 (0.98–1.39)	0.085	1.04 (0.89–1.21)	0.613
Studying full-time	1.22 (1.02–1.45)	0.033	1.21 (1.02–1.43)	0.027
Studying part-time	0.95 (0.82–1.11)	0.548	1.36 (1.17–1.58)	< 0.001
Unemployment or COVID19 benefits	1.17 (0.98–1.41)	0.090	1.04 (0.88–1.23)	0.639
Pension or retirement	1.76 (0.99–3.14)	0.054	0.97 (0.65–1.46)	0.894
Unpaid carer/houseworker	0.99 (0.82–1.19)	0.877	0.98 (0.83–1.16)	0.854
**Completed education**				
Primary education	1.00		1.00	
Secondary education	1.09 (0.74–1.59)	0.671	1.19 (0.87–1.63)	0.273
University education	1.02 (0.70–1.48)	0.926	1.49 (1.09–2.02)	0.012
**Financial problems < 12 months**				
Always/Many times	1.00		1.00	
Some/A few times	1.06 (0.93–1.22)	0.366	0.82 (0.72–0.94)	0.003
Never	1.28 (1.12–1.46)	< 0.001	0.58 (0.51–0.66)	< 0.001

On the other hand, the odds for wanting to learn more about menstruation and the menstrual cycle decreased as age increased (ages 46–55: aOR: 0.22, 95% CI, 0.19–0.26), and when participants reported less financial problems in the last 12 months (e.g., never had financial problems < 12 months: aOR: 0.58, 95% CI, 0.51–0.66). Besides, these odds were significantly lower in participants who identified as trans (aOR: 0.36, 95% CI, 0.24–0.55) and those working full-time (aOR: 0.87, 95% CI, 0.76–0.98). In turn, odds for wanting to have more information were higher in participants studying full-time (aOR: 1.21, 95% CI, 1.02–1.43), and participants with university education (aOR: 1.49, 95% CI, 1.09–2.02) (see Table 3).

### Menstrual poverty and menstrual management

Lifetime experiences of menstrual poverty were disclosed: 22.2% women and PWM reported having had financial problems to afford menstrual products, and 39.9% had experienced difficulties affording preferred menstrual products.

**Fig. 4:**
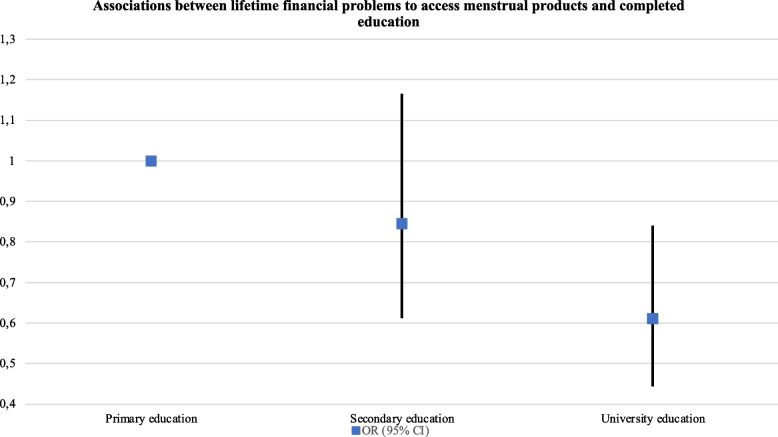
Associations between lifetime financial problems to access menstrual products and completed education

**Fig. 5:**
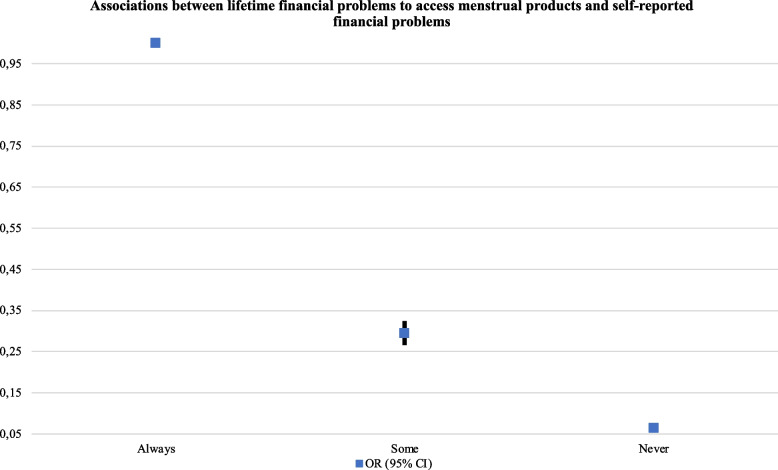
Associations between lifetime financial problems to access menstrual products and self-reported financial problems

The risk of reporting financial problems to access menstrual products increased with age (e.g., participants aged 46–55: aOR: 1.35, 95% CI, 1.14–1.60), among non-binary participants (aOR: 1.67, 95% CI, 1.32–2.11), in those not born in Spain, being the highest among participants born in non-European or Latin American countries (aOR: 2.74, 95% CI, 1.77–4.24), among participants with no permit to reside in Spain (aOR: 4.27, 95% CI, 1.94–9.38), and in participants that were unemployed, receiving a pension or were unpaid carers/houseworkers (e.g., receiving a pension or retired: aOR: 1.59, 95% CI, 1.07–2.37). On the other hand, the risk for menstrual poverty was lower among participants who were self-employed (aOR: 0.71, 95% CI, 0.61–0.83), studying full-time (aOR: 0.82, 95% CI, 0.71-0.095) and had completed university education (aOR: 0.61, 95% CI, 0.44–0.84). Also, the risk for menstrual poverty significantly decreased as participants reported less financial problems in the last 12 months (e.g., never financial problems < 12 months: aOR: 0.06, 95% CI, 0.06–0.07). These associations were similar for the second form of menstrual poverty explored (i.e., hardship to afford preferred menstrual products) (see Table 4 and Figs. 4 and 5).

**Table 4:** Associations between access to menstrual poverty, menstrual management and sociodemographic characteristics (*N* = 20,622)

	**Lifetime financial problems to access menstrual products (** ***N*** ** = 20,518)**		**Lifetime financial problems to choose menstrual products used (** ***N*** ** = 20,467)**		**Overuse menstrual products when no products available (** ***N*** ** = 20,543)**		**Overuse menstrual products when no adequate menstrual management space available (** ***N*** ** = 20,622)**	
	**aOR (95%CI)**	***p*** ** value**	**aOR (95%CI)**	***p*** ** value**	**aOR (95%CI)**	***p*** ** value**	**aOR (95%CI)**	***p*** ** value**
**Age**								
18–25	1.00		1.00		1.00		1.00	
26–35	1.34 (1.18–1.51)	< 0.001	1.11 (1.00–1.23)	0.044	0.71 (0.63–0.80)	< 0.001	0.84 (0.66–1.05)	0.122
36–45	1.28 (1.12–1.46)	< 0.001	0.97 (0.87 (1.08)	0.561	0.47 (0.41–0.53)	< 0.001	0.67 (0.53–0.84)	0.001
46–55	1.35 (1.14–1.60)	< 0.001	0.99 (0.86–1.14)	0.893	0.35 (0.30–0.41)	< 0.001	0.48 (0.36–0.62)	< 0.001
**Gender**								
Women	1.00		1.00		1.00		1.00	
Non-binary/other	1.67 (1.32–2.11)	< 0.001	1.49 (1.21–1.84)	< 0.001	1.25 (0.97–1.59)	0.080	0.77 (0.52–1.13)	0.182
**Trans**								
No	1.00		1.00		1.00		1.00	
Yes	0.91 (0.59–1.41)	0.669	1.10 (0.74–1.64)	0.632	1.30 (0.77–2.18)	0.326	2.57 (0.89–7.45)	0.082
I don’t know	1.03 (0.65–1.63)	0.886	1.08 (0.71–1.64)	0.708	1.18 (0.70–1.97)	0.540	1.92 (0.68–5.43)	0.221
**Place of birth**								
Spain	1.00		1.00		1.00		1.00	
Latin America	1.48 (1.22–1.79)	< 0.001	1.14 (0.96–1.37)	0.142	0.82 (0.68–0.99)	0.044	0.64 (0.46–0.87)	0.005
Europe	1.36 (1.03–1.80)	0.032	1.08 (0.84–1.38)	0.554	0.97 (0.74–1.26)	0.795	1.03 (0.63–1.67)	0.908
Other	2.74 (1.77–4.24)	< 0.001	1.45 (0.96–2.20)	0.079	0.93 (0.58–1.49)	0.765	0.66 (0.31–1.38)	0.270
**Administrative situation**								
Spanish nationality	1.00		1.00		1.00		1.00	
Permanent residency	0.94 (0.74–1.20)	0.625	1.20 (0.97–1.48)	0.098	1.08 (0.86–1.37)	0.500	1.02 (0.68–1.54)	0.912
Temporal residency	1.05 (0.71–1.56)	0.793	1.38 (0.96–1.99)	0.084	1.30 (0.84–2.00)	0.236	1.00 (0.50–2.00)	1.000
No permit/in process	4.27 (1.94–9.38)	< 0.001	2.95 (1.35–6.49)	0.007	0.95 (0.46–1.94)	0.883	0.46 (0.18–1.16)	0.099
**Employment situation**								
Working full-time	1.07 (0.94–1.22)	0.287	1.06 (0.95–1.18)	0.294	1.18 (1.04–1.32)	0.008	1.36 (1.09–1.69)	0.007
Working part-time	1.00 (0.88–1.14)	0.994	1.08 (0.97–1.21)	0.146	1.18 (1.05–1.34)	0.007	1.42 (1.12–1.79)	0.003
Self-employed	0.71 (0.61–0.83)	< 0.001	0.78 (0.69–0.89)	< 0.001	0.97 (0.84–1.11)	0.626	1.00 (0.77–1.29)	0.997
Studying full-time	0.82 (0.71–0.95)	0.010	0.97 (0.85–1.10)	0.589	0.96 (0.82–1.11)	0.552	0.93 (0.71–1.22)	0.585
Studying part-time	1.09 (0.95–1.24)	0.208	1.04 (0.93–1.17)	0.491	1.07 (0.94–1.22)	0.321	1.06 (0.82–1.37)	0.651
Unemployment or COVID19 benefits	1.20 (1.04–1.40)	0.015	1.22 (1.06–1.39)	0.004	1.17 (1.00–1.37)	0.040	1.08 (0.82–1.42)	0.589
Pension or retirement	1.59 (1.07–2.37)	0.021	1.15 (0.81–1.63)	0.441	0.78 (0.54–1.13)	0.193	1.62 (0.71.-3.73)	0.255
Unpaid carer/houseworker	1.19 (1.02–1.40)	0.029	1.23 (1.06–1.41)	0.005	1.04 (0.89–1.22)	0.595	0.98 (0.74–1.29)	0.871
**Completed education**								
Primary education	1.00		1.00		1.00		1.00	
Secondary education	0.84 (0.61–1.16)	0.304	0.88 (0.65–1.18)	0.383	1.29 (0.95–1.76)	0.106	1.86 (1.16–2.98)	0.010
University education	0.61 (0.44–0.84)	0.002	0.70 (0.52–0.94)	0.017	1.20 (0.88–1.63)	0.249	1.87 (1.18–2.99)	0.008
**Financial problems < 12 months**								
Always/Many times	1.00		1.00		1.00		1.00	
Some/A few times	0.29 (0.27–0.33)	< 0.001	0.44 (0.40–0.49)	< 0.001	0.72 (0.64–0.82)	< 0.001	0.67 (0.52–0.86)	0.001
Never	0.06 (0.06–0.07)	< 0.001	0.15 (0.14–0.17)	< 0.001	0.57 (0.50–0.64)	< 0.001	0.48 (0.38–0.61)	< 0.001

As for menstrual products’ cost, 73.5% believed that all menstrual products were very expensive; 23.3% thought that the cost of some products cost  was too high. In order to reduce menstrual poverty, 84.6% of participants believed that menstrual products’ price had to be reduced (e.g., through tax reduction), 62.4% thought that menstrual products should be free or have a reduced price for most-in-need populations, and 34.3% believed that menstrual products should be delivered for free to all women and PWM. Only 0.3% declared that no policies were necessary.

Almost all participants (94.1%) had overused menstrual products when they had no menstrual products available. Many participants (75.2%) had used menstrual products for a longer time than recommended when they could not find adequate menstrual management facilities. Vaginal/vulvar irritation, itchiness or abnormal vaginal discharge were experienced by 75.5% of participants in the 6 months prior to data collection. Among these, 64.4% had not treated the symptoms, 19.7% received treatment from a healthcare professional, and 15% self-treated.

The risk of overusing menstrual products when not having products available was significantly higher in non-binary participants (aOR: 1.25, 95% CI, 0.97–1.59), and those working full-time (aOR: 1.18, 95% CI, 1.04–1.32) or were unemployed or receiving COVID19 benefits (aOR: 1.17, 95% CI, 1.00–1.37). This risk was lowered as age increased (e.g., participants aged 46–55: aOR: 0.35, 95% CI, 0.30–0.40) and financial problems in the last year decreased (e.g., never financial problems < 12 months: aOR: 0.57, 95% CI, 0.50–0.64). As for the risk of overusing menstrual products when adequate menstrual management facilities were unavailable, findings were similar, except for the heightened risk among non-binary participants, and those unemployed or on COVID19 benefits. Besides, the risk increased as educational attainment was higher (e.g., university education: aOR: 1.87, 95% CI, 1.18–2.99). See Table 4 for further details.

### Taboo, embarrassment and experiences of stigma and discrimination

Emotional experiences related to menstruation were mostly “negative” or inferring distress, with participants reporting feeling tired (59.1%), sensitive (58.7%), sad (24.2%), angry (22.7%), dirty (16.5%) and embarrassed (1.8%). It is also important to acknowledge that 14.2% felt happy, 19% calm, 8.4% relieved, and 8.4% felt indifferent when menstruating. Experiences of taboo, embarrassment, stigma and discrimination were common. Most women and PWM (83.3%) disclosed feeling scared of staining their clothes with menstrual blood in public, 58.5% had concealed menstruation, 44.5% had felt discriminated or judged for menstruating, and 19.2% were embarrassed to discuss menstruation.

Participants with higher odds of reporting feeling ashamed of buying or asking menstrual products identified as trans (aOR: 2.51, 95% CI, 1.64–3.83), were born in Latin American (aOR: 1.28, 95% CI, 1.02–1.61) or other non-European countries (aOR: 2.19, 95% CI, 1.41–3.40), did not have Spanish nationality (e.g., no permit/in process: aOR: 3.65, 95% CI, 1.88–7.07), were full-time students (aOR: 1.21, 95% CI, 1.03–1.43), or had completed university education (aOR: 1.70, 95% CI, 1.01–2.86). In turn, these reports were lower as age increased (e.g., participants aged 46–55: aOR: 0.37, 95% CI, 0.30–0.45), and among participants with no reported financial difficulties in the year preceding the study (aOR: 0.82, 95% CI, 0.71–0.93) (Table 5).

**Table 5:** Associations between menstrual-related taboo, stigma, discrimination and sociodemographic characteristics (*N* = 20,619)

	**Shame buying or asking for menstrual products (** ***N*** ** = 20,619)**		**Fear of staining in public (** ***N*** ** = 20,196)**		**Has concealed menstruation (N = 19,944)**		**Shame discussing menstruation (** ***N*** ** = 20,159)**		**Experiences of discrimination or judgment due to menstruating (** ***N*** ** = 19,602)**	
	**aOR (95%CI)**	***p*** ** value**	**aOR (95%CI)**	***p*** ** value**	**aOR (95%CI)**	***p*** ** value**	**aOR (95%CI)**	***p*** ** value**	**aOR (95%CI)**	***p*** ** value**
**Age**										
18–25	1.00		1.00		1.00		1.00		1.00	
26–35	0.59 (0.52–0.67)	< 0.001	0.62 (0.54–0.71)	< 0.001	0.83 (0.75–0.92)	< 0.001	0.81 (0.72–0.91)	< 0.001	1.02 (0.92–1.12)	0.738
36–45	0.46 (0.40–0.53)	< 0.001	0.54 (0.46–0.62)	< 0.001	0.80 (0.72–0.89)	< 0.001	0.82 (0.72–0.93)	0.002	0.83 (0.75–0.92)	< 0.001
46–55	0.37 (0.30–0.45)	< 0.001	0.56 (0.47–0.67)	< 0.001	0.71 (0.63–0.82)	< 0.001	0.75 (0.63–0.88)	< 0.001	0.54 (0.47–0.62)	< 0.001
**Gender**										
Women	1.00		1.00		1.00		1.00		1.00	
Non-binary/other	1.21 (0.91–1.59)	0.180	0.88 (0.68–1.15)	0.346	1.38 (1.11–1.71)	0.003	1.08 (0.85–1.37)	0.542	1.88 (1.52–2.33)	< 0.001
**Trans**										
No	1.00		1.00		1.00		1.00		1.00	
Yes	2.51 (1.64–3.83)	< 0.001	1.18 (0.67–2.06)	0.565	1.48 (0.96–2.27)	0.076	2.37 (1.59–3.54)	< 0.001	1.10 (0.73–1.66)	0.653
I don’t know	1.19 (0.71–1.98)	0.507	1.18 (0.67–2.07)	0.576	1.08 (0.71–1.65)	0.708	1.38 (0.89–2.14)	0.156	1.50 (0.97–2.32)	0.070
**Place of birth**										
Spain	1.00		1.00		1.00		1.00		1.00	
Latin America	1.28 (1.02–1.61)	0.036	1.05 (0.83–1.32)	0.695	1.10 (0.92–1.31)	0.283	1.18 (0.97–1.44)	0.103	0.95 (0.80–1.13)	0.535
Europe	1.28 (0.94–1.74)	0.120	1.08 (0.79–1.48)	0.636	1.08 (0.85–1.37)	0.543	1.48 (1.14–1.93)	0.003	1.30 (1.02–1.66)	0.033
Other	2.19 (1.41–3.40)	0.001	1.00 (0.57–1.77)	0.993	1.41 (0.92–2.16)	0.111	2.17 (1.44–3.28)	< 0.001	1.31 (0.87–1.98)	0.195
**Administrative situation**										
Spanish nationality	1.00		1.00		1.00		1.00		1.00	
Permanent residency	1.42 (1.09–1.86)	0.010	0.97 (0.74–1.27)	0.818	0.94 (0.77–1.16)	0.577	1.10 (0.87–1.39)	0.446	0.78 (0.63–0.96)	0.019
Temporal residency	1.69 (1.13–2.53)	0.011	1.44 (0.83–2.50)	0.201	1.29 (0.88–1.88)	0.189	1.48 (1.01–2.14)	0.042	0.89 (0.62–1.27)	0.514
No permit/in process	3.65 (1.88–7.07)	< 0.001	2.67 (0.81–8.82)	0.107	2.04 (1.01–4.13)	0.047	2.11 (1.10–4.03)	0.024	0.62 (0.32–1.17)	0.140
**Employment situation**										
Working full-time	0.93 (0.80–1.07)	0.306	1.15 (1.00–1.31)	0.046	1.11 (1.00–1.23)	0.048	1.07 (0.94–1.21)	0.303	0.98 (0.88–1.09)	0.748
Working part-time	0.93 (0.80–1.07)	0.302	0.98 (0.85–1.13)	0.797	1.07 (0.96–1.19)	0.208	0.91 (0.80–1.03)	0.156	1.07 (0.96–1.19)	0.194
Self-employed	0.89 (0.73–1.07)	0.207	0.74 (0.64–0.87)	< 0.001	0.90 (0.79–1.01)	0.077	0.94 (0.81–1.09)	0.428	1.06 (0.93–1.20)	0.381
Studying full-time	1.21 (1.03–1.43)	0.019	1.45 (1.21–1.74)	< 0.001	1.25 (1.11–1.42)	< 0.001	1.29 (1.12–1.49)	< 0.001	1.31 (1.16–1.48)	< 0.001
Studying part-time	1.01 (0.86–1.18)	0.920	0.87 (0.76–1.00)	0.054	0.99 (0.89–1.11)	0.858	1.06 (0.93–1.20)	0.419	1.28 (1.14–1.43)	< 0.001
Unemployment or COVID19 benefits	0.99 (0.83–1.20)	0.951	1.07 (0.90–1.27)	0.462	1.17 (1.03–1.34)	0.017	0.99 (0.85–1.16)	0.907	1.21 (1.06–1.38)	0.004
Pension or retirement	0.91 (0.56–1.49)	0.713	0.90 (0.58–1.42)	0.660	1.07 (0.76–1.51)	0.700	1.05 (0.70–1.57)	0.825	1.18 (0.84–1.67)	0.346
Unpaid carer/houseworker	0.90 (0.73–1.11)	0.311	0.85 (0.72–1.01)	0.057	0.88 (0.76–1.00)	0.054	1.02 (0.86–1.20)	0.813	1.30 (1.13–1.49)	< 0.001
**Completed education**										
Primary education	1.00		1.00		1.00		1.00		1.00	
Secondary education	1.38 (0.82–2.32)	0.231	1.15 (0.80–1.65)	0.443	1.56 (1.16–2.08)	0.003	1.09 (0.74–1.63)	0.656	1.58 (1.15–2.17)	0.005
University education	1.70 (1.01–2.86)	0.045	1.19 (0.83–1.71)	0.335	2.22 (1.67–2.97)	< 0.001	1.65 (1.11–2.44)	0.013	2.10 (1.53–2.88)	< 0.001
**Financial problems < 12 months**										
Always/Many times	1.00		1.00		1.00		1.00		1.00	
Some/A few times	0.92 (0.80–1.05)	0.207	0.98 (0.86–1.12)	0.765	1.03 (0.94–1.14)	0.518	0.96 (0.86–1.08)	0.531	0.70 (0.63–0.77)	< 0.001
Never	0.82 (0.71–0.93)	0.003	0.78 (0.69–0.89)	< 0.001	0.85 (0.77–0.93)	0.001	0.85 (0.76–0.95)	0.006	0.46 (0.42–0.51)	< 0.001

**Fig. 6:**
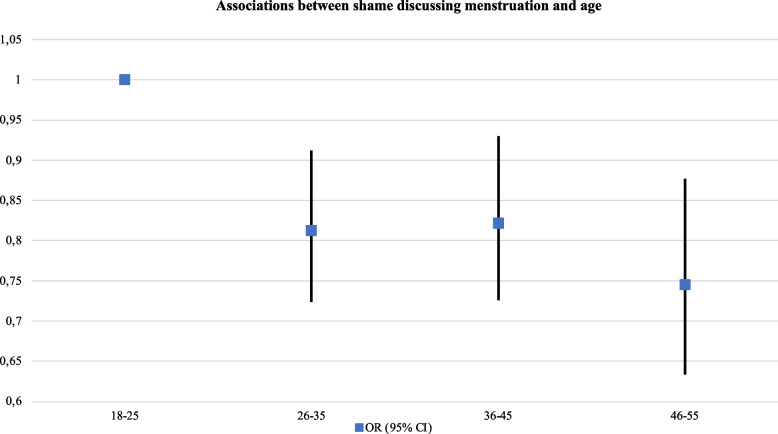
Associations between shame discussing menstruation and age

**Fig. 7:**
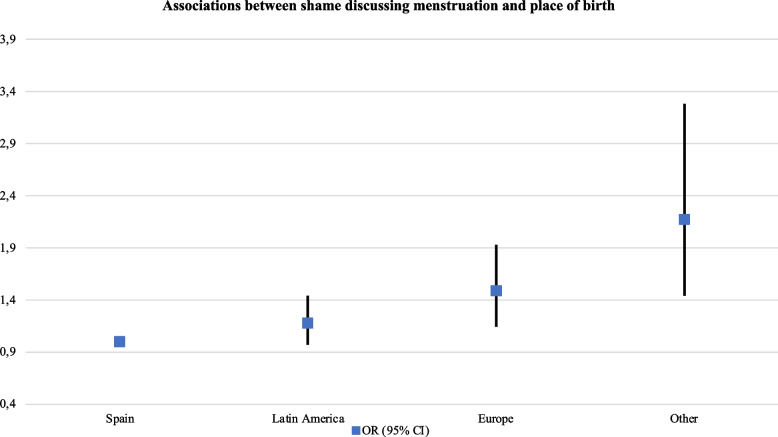
Associations between shame discussing menstruation and place of birth

The report of taboo, stigma and experiences of discrimination also decreased with age and as financial problems lessened. However, it increased in full-time students and participants with completed university education (see Table 5). Non-binary participants were at higher risk to report having concealed menstruation (aOR: 1.38, 95% CI, 1.11–1.71) and having experienced discrimination or judgment due to menstruating (aOR: 1.88, 95% CI, 1.52–2.33). Trans people were also at a heightened risk of reporting shame to discuss menstruation (aOR: 2.37, 95% CI, 1.59–3.54). This was also higher among women and PWM born in non-European or Latin American countries (aOR: 2.17, 95% CI, 1.44–3.28) (Figs. 6 and 7 ).

The odds for reporting experiences of menstrual discrimination/judgment were higher among participants born in European countries other than Spain (aOR: 1.30, 95% CI, 1.02–1.66). Also, in participants with a more vulnerable administrative situation (e.g., no residency permit: aOR: 2.11, 95% CI, 1.10–4.03). Working full-time was associated with a higher risk of fearing staining in public with menstrual blood (aOR: 1.15, 95% CI, 1.00–1.31) and concealing menstruation (aOR: 1.11, 95% CI, 1.00–1.23). On the other hand, the risk of fearing staining was significantly lower in women and PWM who were self-employed (aOR: 0.74, 95% CI, 0.64–0.87), or were unpaid carers or houseworkers (aOR: 0.85, 95% CI, 0.72–1.01). See Table 5 for more details.

### Social, community and economic participation

Feeling less able to pay attention or cope with day-to-day issues (73.1%) and perceiving oneself as less productive (70.1%) when menstruating was common among participants. Interestingly, 13.1% reported not having paid attention to their levels of productivity while menstruating.

The risk for reporting feeling less able to cope with day-to-day activities and decreased productivity was significantly lower as age increased [e.g., participants aged 46–55: aOR: 0.32, 95% CI, 0.28–0.37; aOR: 0.25, 95% CI, 0.21–0.30] and financial problems lessened [e.g., no financial problems < 12 months: aOR: 0.47, 95% CI, 0.42–0.53; aOR: 0.53, 95% CI, 0.46–0.62]. The odds were also lower among participants working full-time [aOR: 0.74, 95% CI, 0.65–0.84; aOR: 0.66, 95% CI, 0.57–0.77]. Instead, the odds were significantly higher among women and PWM with university education [aOR: 1.63, 95% CI, 1.19–2.21; aOR: 1.59, 95% CI, 1.10–2.28]. Furthermore, the risk for feeling less able to cope with day-to-day tasks was significantly higher among non-binary participants (aOR: 1.36, 95% CI, 1.05–1.77), and participants studying full-time (aOR: 1.44, 95% CI, 1.23–1.68). Participants with no permit to reside in Spain presented significantly lower odds to report decreased productivity when menstruating (aOR: 0.41, 95% CI, 0.19–0.86) (see Table 6).

**Table 6:** Associations between social, community and economic participation, and sociodemographic characteristics (N = 20,622)

	**Feel less able to cope with day-to-day activities during menstruation (** ***N*** ** = 19,997)**		**Feels less productive during menstruation (** ***N*** ** = 17,330)**		**Work absenteeism during menstruation (** ***N*** ** = 18,463)**		**Education absenteeism during menstruation (** ***N*** ** = 18,271)**		**Decline social meetings during menstruating (** ***N*** ** = 20,182)**	
	**aOR (95%CI)**	***p*** ** value**	**aOR (95%CI)**	***p*** ** value**	**aOR (95%CI)**	***p*** ** value**	**aOR (95%CI)**	***p*** ** value**	**aOR (95%CI)**	***p*** ** value**
**Age**										
18–25	1.00		1.00		1.00		1.00		1.00	
26–35	0.78 (0.68.0.88)	< 0.001	0.76 (0.65–0.89)	0.001	1.06 (0.93–1.20)	0.375	0.88 (0.79–0.98)	0.024	0.99 (0.90–1.09)	0.846
36–45	0.56 (0.49–0.63)	< 0.001	0.50 (0.42–0.58)	< 0.001	0.93 (0.82–1.07)	0.307	0.51 (0.45–0.57)	< 0.001	0.73 (0.66–0.82)	< 0.001
46–55	0.32 (0.28–0.37)	< 0.001	0.25 (0.21–0.30)	< 0.001	0.91 (0.76–1.07)	0.254	0.33 (0.29–0.38)	< 0.001	0.48 (0.48–0.55)	< 0.001
**Gender**										
Women	1.00		1.00		1.00		1.00		1.00	
Non-binary/other	1.36 (1.05–1.77)	0.020	1.06 (0.78–1.43)	0.711	1.65 (1.30–2.08)	< 0.001	1.57 (1.23–2.00)	< 0.001	1.70 (1.37–2.11)	< 0.001
**Trans**										
No	1.00		1.00		1.00		1.00		1.00	
Yes	1.51 (0.82–2.79)	0.184	1.46 (0.72–2.98)	0.294	1.01 (0.62–1.66)	0.970	0.88 (0.54–1.41)	0.588	0.87 (0.57–1.30)	0.490
I don’t know	1.15 (0.67–1.99)	0.611	0.82 (0.45–1.50)	0.513	1.25 (0.78–2.03)	0.355	0.71 (0.45–1.13)	0.153	0.78 (0.51–1.17)	0.230
**Place of birth**										
Spain	1.00		1.00		1.00		1.00		1.00	
Latin America	0.98 (0.81–1.20)	0.880	0.95 (0.74–1.21)	0.655	1.24 (1.01–1.53)	0.037	0.75 (0.62–0.90)	0.002	0.96 (0.81–1.15)	0.675
Europe	1.55 (1.16–2.06)	0.003	1.34 (0.94–1.90)	0.100	1.19 (0.90–1.59)	0.223	1.32 (1.01–1.73)	0.039	1.22 (0.96–1.55)	0.098
Other	1.04 (0.63–1.71)	0.877	0.98 (0.52–1.85)	0.958	1.40 (0.85–2.30)	0.184	1.13 (0.71–1.80)	0.602	1.64 (1.07–2.52)	0.023
**Administrative situation**										
Spanish nationality	1.00		1.00		1.00		1.00		1.00	
Permanent residency	0.92 (0.72–1.18)	0.520	1.05 (0.77–1.42)	0.771	0.99 (0.77–1.27)	0.957	0.76 (0.61–0.95)	0.016	0.82 (0.67–1.01)	0.064
Temporal residency	0.88 (0.56–1.38)	0.575	0.82 (0.48–1.40)	0.465	1.34 (0.89–2.03)	0.160	0.82 (0.55–1.20)	0.303	0.75 (0.52–1.06)	0.106
No permit/in process	1.56 (0.64–3.81)	0.330	0.41 (0.19–0.86)	0.019	3.30 (1.66–6.55)	0.001	1.34 (0.63–2.85)	0.450	1.09 (0.56–2.13)	0.791
**Employment situation**										
Working full-time	0.74 (0.65–0.84)	< 0.001	0.66 (0.57–0.77)	< 0.001	0.62 (0.55–0.71)	< 0.001	0.75 (0.66–0.84)	< 0.001	0.81 (0.73–0.89)	< 0.001
Working part-time	0.83 (0.73–0.94)	0.004	0.74 (0.63–0.87)	< 0.001	0.58 (0.51–0.66)	< 0.001	0.85 (0.76–0.96)	0.006	0.81 (0.73–0.91)	< 0.001
Self-employed	0.89 (0.77–1.02)	0.094	0.84 (0.70–1.00)	0.046	1.40 (1.22–1.61)	< 0.001	0.88 (0.77–1.00)	0.057	0.87 (0.77–0.98)	0.021
Studying full-time	1.44 (1.23–1.68)	< 0.001	1.20 (0.98–1.46)	0.073	1.15 (0.99–1.35)	0.070	1.55 (1.35–1.78)	< 0.001	1.17 (1.03–1.32)	0.013
Studying part-time	1.29 (1.12–1.48)	< 0.001	1.11 (0.94–1.31)	0.214	1.11 (0.97–1.27)	0.114	1.68 (1.49–1.91)	< 0.001	1.18 (1.05–1.31)	0.004
Unemployment or COVID19 benefits	0.97 (0.83–1.14)	0.697	0.86 (0.71–1.04)	0.121	0.77 (0.66–0.90)	0.001	1.05 (0.90–1.21)	0.553	1.08 (0.95–1.23)	0.232
Pension or retirement	0.91 (0.61–1.36)	0.648	0.92 (0.57–1.48)	0.720	1.13 (0.71–1.79)	0.616	1.28 (0.86–1.91)	0.227	1.26 (0.88–1.78)	0.202
Unpaid carer/houseworker	0.93 (0.79–1.09)	0.355	0.89 (0.73–1.08)	0.226	0.97 (0.82–1.14)	0.685	0.92 (0.78–1.07)	0.259	0.94 (0.82–1.08)	0.402
**Completed education**										
Primary education	1.00		1.00		1.00		1.00		1.00	
Secondary education	1.22 (0.89–1.66)	0.213	1.35 (0.94–1.95)	0.108	0.88 (0.61–1.27)	0.492	1.31 (0.92–1.85)	0.133	1.30 (0.97–1.74)	0.077
University education	1.63 (1.19–2.21)	0.002	1.59 (1.10–2.28)	0.013	0.89 (0.62–1.27)	0.518	1.74 (1.23–2.46)	0.002	1.39 (1.04–1.85)	0.025
**Financial problems < 12 months**										
Always/Many times	1.00		1.00		1.00		1.00		1.00	
Some/A few times	0.73 (0.64–0.83)	< 0.001	0.78 (0.67–0.92)	0.002	0.80 (0.72–0.90)	< 0.001	0.79 (0.70–0.88)	< 0.001	0.78 (0.71–0.87)	< 0.001
Never	0.47 (0.42–0.53)	< 0.001	0.53 (0.46–0.62)	< 0.001	0.55 (0.49–0.61)	< 0.001	0.51 (0.46–0.57)	< 0.001	0.51 (0.46–0.56)	< 0.001

Work absenteeism was disclosed by 20.3% of women and PWM who were working at the time of data collection. Among students, 62.7% did not attend their school/university when menstruating. Just over half (53.8%) reported declining social plans, 79.7% did not practice physical activity, and 72.3% did not go to the pool/beach when menstruating. The reasons to stop doing these activities were: dysmenorrhea (68.6%), tiredness (65.3%), emotional status (48.2%), abundant bleeding (32.4%) and the need to conceal menstruation (0.9%). Participants reported teleworking or timetable flexibility (76.4%) and the menstrual leave (49.9%) as preferred policies to improve menstrual management in workplaces. These measures were not deemed necessary by 11.4% of the participants.

**Fig. 8:**
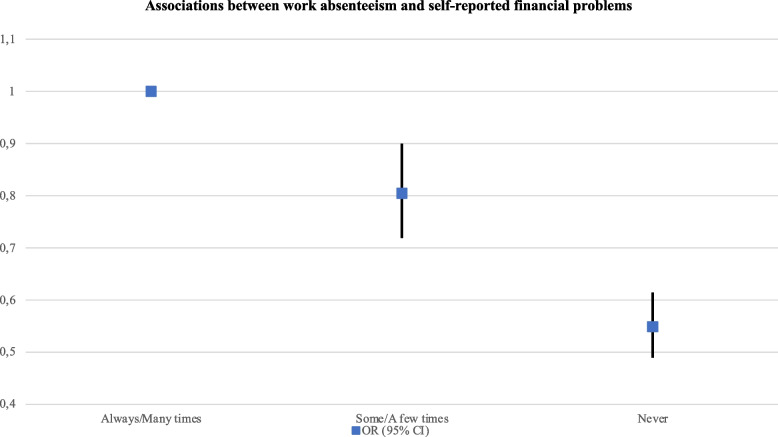
Associations between work absenteeism and self-reported financial problems

The odds for work absenteeism were significantly higher among non-binary menstruators (aOR: 1.65, 95% CI, 1.30–2.08), participants with no residency permit (aOR: 3.30, 95% CI, 1.66–6.55), and women and PWM who were self-employed (aOR: 1.40, 95% CI, 1.22–1.61). On the other hand, the risk for work absenteeism was significantly lower among participant working full-time (aOR: 0.62, 95% CI, 0.55–0.71) and those having some/a few (aOR: 0.80, 95% CI, 0.72–0.90) or no (aOR: 0.55, 95% CI, 0.49–0.61) financial problems in the previous year (see Table 6) (Fig. 8).

The risk for education absenteeism during menstruation was significantly higher in non-binary participants (aOR: 1.57, 95% CI, 1.23–2.00) and participants with completed university education (aOR: 1.74, 95% CI, 1.23–2.46). The odds of education absenteeism were significantly lower as age increased (e.g., participants aged 46–55: aOR: 0.33, 95% CI, 0.29–0.38) and financial problems lessened (e.g., no financial problems < 12 months: aOR: 0.51, 95% CI, 0.46–0.57) (see Table 6).

The odds for declining social meetings during menstruation were significantly higher among participants who identified as non-binary (aOR: 1.70, 95% CI, 1.37–2.11), and born in non-European or Latin American countries (aOR: 1.64, 95% CI, 1.07–2.52). Also, among full-time students (aOR: 1.17, 95% CI, 1.03–1.32) and women and PWM with university education (aOR: 1.39, 95% CI, 1.04–1.85). The odds were significantly lower as age increased (e.g., participants aged 46–55: aOR: 0.48, 95% CI, 0.48–0.55) and participants reported fewer financial problems (e.g., no financial issues < 12 months: aOR: 0.51, 95% CI, 0.46–0.56). See Table 6 for more information.

## Discussion

This study aimed to describe menstrual inequities and their associations with sociodemographic factors, among women and PWM aged 18–55 in Spain. Menstrual inequities were widespread in our sample, having a more profound impact on some groups of women and PWM, such as those with less socioeconomic resources. Other main associations were between menstrual inequities and completed education, employment and administrative status, and non-binary or trans identification. Main findings are discussed below.

### Access to healthcare services for menstrual health

Access to menstrual-related healthcare services is a core element for menstrual equity, also considering that women have been reported to have more difficulties in accessing healthcare compared to men [36]. Although more than half of the participants had sought professional assistance for menstruation, a significant number of them did not think it was necessary to access healthcare services for menstruation. As expected, lifetime access to healthcare services was higher in older participants as they had had more time to access and potentially more opportunities for consultations. The fact that participants receiving a pension or those who were retired also had higher odds of accessing healthcare services for menstruation might be explained by the potentially higher age of this participant group, or due to experiencing less time poverty [37]. Participants on health benefits could have reported more access to healthcare services due to poorer health. The odds of healthcare access were higher in employed and self-employed participants. This can be potentially related to the age of women and PWM in these groups (e.g., they may be at the time of trying to get pregnant). This association could also be explained by poorer menstrual health outcomes of workers and self-employed women and PWM, as their access to the labor force may increase stress and burden of care [38].

Education is an important social determinant of health [39, 40]. Consistently with previous evidence on the link between education, health outcomes and health equity [41, 42], completing university education was a protective factor for healthcare access. Women and PWM with higher education may be more informed, given their increased access to educational resources. At the same time, participants with a higher educational attainment might have been more empowered, less impacted by time poverty [37] and have a higher health and body literacy. The latter has been previously linked to positive health outcomes [43]. Our findings also reveal a link between financial constraints and an increased access to healthcare services, possibly due to poorer menstrual health [10]. However, special attention should be paid to women and PWM with a lower socioeconomic status, as previous reports report the increased barriers for healthcare access among socioeconomically deprived populations [36]. The link with administrative status is not clear, although our data suggests that not having the Spanish nationality may be associated with a decreased access to healthcare services for menstruation. This is coherent with previous evidence on, not only the legislative, financial and economic barriers that migrant populations face to access healthcare services [36, 44], but also institutionalized racism and the lack of cultural safety in healthcare systems [45, 46]. Especially migrant women and PWM with no access to a public health insurance card may face significant barriers to healthcare access.

### Access to menstrual education and knowledge

The number of women and PWM that did not have enough information on the menstrual cycle and menstruation before menarche was reasonably high (45.3%), with 12.5% not knowing what menstruation was at menarche. Furthermore, around half were not feeling ready to menstruate at the time of menarche. Not only menstrual education is essential [47, 48], but how girls and young PWM feel about menstruating, and the resources they are equipped with to manage menstruation and the social changes that commonly come with menarche [29, 49]. As apparent in our study, most participants learnt from their families, school or friends at the time of menarche. Instead, the internet and social media appear to become main sources of menstrual information during adulthood. This points to the role of social media and online networks on public health strategies [50, 51]. Also, to its potential drawbacks of being sources of “infodemics” [52] and generating further social inequities as a result of the digital divide [53], among other challenges [54]. However, these data can serve to support and inform the use of online networks and platforms in strategies that promote menstrual education and equity.

Being older was a risk factor for not having had access to menstrual education pre-menarche. This is expected given that reproductive and sexual health curricula (where menstrual education is usually included) has started to be particularly present in young people’s education in the last few years [55]. Although this could be attributed to recall biases, the access to information through information and communication technology, and the influence the feminist movement has had in the last few years may have also led to an increased access to menstrual education amongst. Spain has experienced some profound socio-political changes in the last decades, which can also explain our findings. Spain was under a dictatorship from 1939 until 1975, which led, not only a systematic violation of human rights, but a profound regression in the educational system. The Francoist dictatorship was characterized for being an authoritarian regime that enforced Catholicism. Women lost many human and civil rights that they had been granted to at the start of the twentieth century, which included the prohibition of the use of contraception, abortion, and divorce by law. The post-dictatorship decades have led to a slow restructuring of our political and societal systems, which have also led to including sexual and reproductive education in the school curricula. Considering that many of our participants grew up in the last decade of Franco’s dictatorship, and during the transition to democracy, it is plausible that they received limited to no menstrual education pre-menarche.

Participants born in non-European or Latin American countries were also found to have had less access to menstrual education pre-menarche. This indicates disparities in the access to menstrual education across countries and world regions. On the other hand, trans people in our study appeared to be less interested in learning about menstruation and the menstrual cycle, possibly given the potential distress and gender dysphoria that some trans people may experience related to menstruation [56–59].

### Menstrual poverty and menstrual management

Women and PWM in our study reported a lifetime prevalence of menstrual poverty between 22.2% (to afford any menstrual product) and 39.9% (to afford preferred menstrual products). Although evidence is still scarce, these estimates are higher than those reported in previous studies. Menstrual poverty in young people has been calculated to be around 10% in the UK [32] and between 10–14.2% in the US [21] in young populations. Spanish national statistics indicate that 10.9% of employed women, 31.2% of unemployed women and 42.6% of women receiving unemployment benefits lived in a situation of relative poverty in 2020 [60]. These reports are consistent with our estimates of menstrual poverty. However, the prevalence of menstrual poverty in Spain may be higher as our sample was fairly socioeconomically privileged. This is a limitation in our research that may have led to underestimate, not only realities of menstrual poverty but menstrual inequity. However, our study suggests that menstrual poverty may not only affect women and PWM living in situations of poverty, but other women that may experience occasional financial hardship. This point leads us to mention how common precarious work is in Spain, with poorly paid, insecure and temporary jobs having particularly increased since the 2008 economic crisis, and during the COVID19 syndemic. Also, to highlight that poverty and precariousness are gendered, highly affecting women and gender non-conforming people [61, 62]. In fact, our study shows that non-binary people are more at risk of menstrual poverty. Besides, menstrual poverty risk was higher in migrant populations, potentially those more socioeconomically deprived. As for the relationship between menstrual poverty and age, increased age was another risk factor for menstrual poverty in our study. However, this should be interpreted with caution as data were collected for lifetime experiences of menstrual poverty. However, there may be generational differences that are worth exploring in future research.

On the other hand, menstrual management is imperative for women and PWM’s health and wellbeing [11, 12, 63–66]. Unsafe management of menstruation and overusing menstrual products may lead to several health problems, such as lower reproductive tract infections [67, 68]. Most of the participants in our study had overused menstrual products, with 75.5% experiencing symptoms related to the use of menstrual products in the six months preceding the study. Product overuse may be one of the reasons for such a high prevalence of menstrual product-related symptoms identified in our research, together with the lack of access to adequate menstrual management facilities. The odds for overusing menstrual products were higher in non-binary participants, although not related to the lack of access to menstrual management facilities. It also affected employed participants more, particularly as the challenges to manage menstruation are greater if working away from home due to gender discrimination in urban and built environments [69]. Besides, a link between financial hardship and the overuse of menstrual products was found. This suggests that socioeconomically deprived women and PWM are at higher risk for using menstrual products for longer than recommended, which may be accompanied with an increase in (menstrual) health risks. At the same time, higher educational attainment was a risk factor for menstrual product overuse. As already discussed, participants with university studies may be more empowered and have less restrictions (e.g., time poverty) to identify and report some menstrual inequity-related experiences.

### Taboo, embarrassment and experiences of stigma and discrimination

Consistently with previous evidence [13, 70, 71], menstrual-related taboo, embarrassment and experiences of stigma and discrimination were significantly common among women and PWM in our sample. Being employed or studying were risk factors to report menstrual-related taboo and embarrassment, which links to the challenges of managing menstruation in workplaces [26] and educational [72] settings. Another risk factor was having completed university education. In order to understand this finding, first we need to consider that most participants had completed higher education. Also, that the relationship between education and the report of taboo, embarrassment, stigma and discrimination may be mediated by having more resources to identify and report such experiences. It may also be that women and PWM with higher education attainment are less burdened with day-to-day hardships, allowing them to pay more attention to experiences of menstrual taboo, stigma and discrimination. On the contrary and coherently with other findings from our research, having less or no financial problems was a protective factor for menstrual-related taboo, embarrassment and experiences of stigma and discrimination.

The odds to report menstrual-related embarrassment were higher among trans people. Likewise, the odds for concealing menstruation and experiencing menstrual-related stigma and discrimination increased in non-binary participants. This may be associated with the prevalent stigma and discrimination that gender non-conformity people experience, including related to menstruation [56–59] and in healthcare settings [73, 74].

There was also a tendency for some migrant populations to be more at risk of reporting taboo, stigma and discrimination. While this may be related to sociocultural conceptualisations of menstruation, it is essential to highlight that migrant populations, and especially racialised individuals or those in a situation of socioeconomic deprivation, are already a target of structural stigma and discrimination. Prevailing experiences of stigma and discrimination can have a profound impact on social, psychological [75] and physical wellbeing [76, 77], thus research and sociopolitical actions on menstrual inequity should pay particular attention to the structural violence  that migrant populations experience.

### Social, community and economic participation

It was common for women and PWM in our study to report feeling less able to cope with day-to-day activities and be productive when menstruating. Although this is not problematic per se, it leads us to question whether women and PWM count with enough resources to practice self-care to manage menstruation. Participants who reported being more burdened by menstruation in their day-to-day were non-binary participants, younger people, and women and PWM with more financial problems. As already discussed, this points towards the need to particularly address menstrual inequities among gender non-conformity individuals and those more socioeconomically deprived. Further, being employed was found to be a protective factor, which could be explained by the fact that, often, employees cannot avoid productive work. This may particularly affect women and PWM with precarious working conditions or experiencing higher economic hardship [62].

Menstruation-related work and education absenteeism were high. Based on participants’ responses and previous research [25], it is urgent to support menstrual management policies in work and educational environments. Future research should further investigate the strategies that may be most relevant to women and PWM, and those most feasible to implement, to then inform policymaking processes. Also, to further evaluate the impact of education absenteeism on women and PWM. Policies should not only focus on supporting self-care and menstrual management [26] but also address the reasons why women and PWM might need to be absent from work or their studies during menstruation. Non-binary people and some migrant groups were again found to be more affected. It is however imperative that these findings are not used to discriminate against these groups, but rather promote policies that can be adapted to the needs of different populations. Understandably, experiencing financial hardship was a protective factor for work absenteeism, as the socioeconomic costs of absenteeism could be certainly greater.

Lastly, social and community participation must not be disregarded, as our study revealed that it can be greatly compromised during menstruation. The benefits of social and community participation have already been examined in previous research [78, 79], being fundamental elements to attain social and health equity [80]. Encouraging women and PWM’s social and community participation, while promoting self-care and menstrual management, can be core pillars towards menstrual equity.

### Strengths, limitations and reflections

The main strength of this study is that it provides, to our knowledge, the first comprehensive description of menstrual inequities in Spain. A second strength is the inclusion of a large number of women and PWM across the Spanish territory. There were also limitations. First, the questionnaire used for data collection took around 20 minutes to complete. This may have led to drop-outs and the refusal to participate. Despite the efforts to design an accessible questionnaire, the team is also aware that the complexity of some questions may have also had a negative impact on participation. Besides, based on its methodological nature, this study cannot be representative to the whole menstruating population in Spain, as we used an online questionnaire and sampling was non-probabilistic. As already presented in the Results section, our sample differs from available representative data in Spain in age distribution, completed education, the proportion of migrant populations, and people who were unemployed and those receiving pensions/benefits. Our sample included a higher proportion of younger people who had completed university studies, were born in Spain and were not unemployed or receiving a pension or State benefits. However, representative data on employed populations and financial satisfaction were similar to the data in our study. Thus, overall, we can conclude that our participants were in a relatively socioeconomically privileged situation. This suggests that our study may be underestimating menstrual inequities. Besides, it is important to consider this information when interpreting the data from our study. Although results should be interpreted with caution, the sample size is large and representative enough to present a good estimate of menstrual inequity in Spain. Moreover, we need to acknowledge the potential detrimental impact of the digital divide on the recruitment process and representation of the sample. As noticeable from our results, over 60% of participants had completed university studies and over half were employed. Thus, our findings may not be representative of populations with less access to social and economic resources. Acknowledging this limitation is important as this study may have underestimated menstrual inequities in Spain. We may have also missed understanding the profound impact of menstrual inequity among socioeconomically vulnerable and hard-to-reach populations (e.g., those living in situations of homelessness). Despite this limitation may challenge the interpretation of our findings, the data presented in this publication present a first approximation to describing menstrual inequities in Spain. The team is committed to continuing this line of research to offer an overview of menstrual inequities that is representative, considers diversities and reaches out to those most vulnerable to experiencing menstrual inequities.

Moreover, the use of self-reported data entails further challenges to interpret our findings. One of the limitations of self-reported data is the inability to understand participants' experiential knowledge and subjective experience. Thus, the research team cannot be sure of how participants have understood and answered some questions (e.g., regarding “feeling less productive”). Although this could have had an impact on our results, as these may have not accounted for subjective diversity, the team assumes this challenge and calls for a mindful interpretation of our results. The research team has conducted qualitative research, which complements and expands the findings provided in this manuscript; this could partially address the limitations of quantitative self-reported methods. The use of self-reported data in inequity research presents another challenge. As other researchers have previously identified and discussed [81], we are limited to understand how the response to certain variables (e.g., menstrual poverty) may differ by socioeconomic proxies (e.g., completed education). This presents challenges to describe and estimate menstrual inequities, entailing a potential under or overestimation of these inequities. Further research is required to improve data collection and interpreting methods that account for social inequities, such as for differences in self-report reporting by educational level [81].

As a final point, data were collected at a time when tax reductions for menstrual products and a new (and polemic) law on Trans Rights were being discussed at the Spanish Congress. We believe that collecting data in this context has had a two-fold impact on recruitment and data collection. While the study was highly disseminated given the public discussions around menstrual health and equity at the time of data collection, we are aware that some women refused to participate in the study as it was also welcoming non-binary and trans people who menstruate to participate. Additionally, using gender-neutral/inclusive language in Spanish is difficult given that grammatical gender is pervasive and there is no neutral grammatical gender in common language use. In order to make the questionnaire accessible to most participants, the questionnaire’s language was mostly using “feminine” language structures in places where gender-neutral/inclusive language could not be used. Some non-binary and trans people may have refused to participate for this reason, as they may have not felt represented. In spite of this limitation, the sample of non-binary and trans people represented 4% of the total sample (3.4% and 0.8% respectively). There are no estimates of how many non-binary and trans people live in Spain. However, estimates in the United States indicate that 0.3–4.5% of adults identify as trans and/or gender diverse [82, 83]. Based on this, we could have an acceptable representation of non-binary and trans people in our study.

### Implications for research and policy

Recommendations for future research and policy development can be drawn from this study. First, research actions could examine the impact of menstrual inequities on women and PWM’s emotional and physical health, including the potential associations between menstrual inequities and (menstrual) health outcomes. Future studies could also focus on younger populations, and on particular forms of menstrual inequities (e.g., access to healthcare services for menstruation). Developing a menstrual inequity index could be another significant contribution to the current body of research. Second, policy strategies should be multifaceted and multidimensional, addressing menstrual inequities comprehensively rather than only focusing on, for instance, tackling menstrual poverty. As for menstrual poverty, it should not only be considered as not being able to afford menstrual products, but also as not being able to choose preferred menstrual product(s) due to financial constraints. Taxes on menstrual products need to be reduced, not just to improve affordability but as a matter of social justice. Given that tax reduction will not be sufficient to tackle menstrual poverty, policies should be far-reaching and intersectoral. Policies to ensure affordability and availability among socioeconomically deprived women and PWM could have a positive impact. Together with menstrual poverty strategies, policies should ensure formal and community-based menstrual education aimed at younger and older generations. Access to menstrual health services should also be ensured, particularly through educating the population and healthcare professionals on menstrual health. Healthcare services also need to be structured mindfully around the needs and barriers faced by non-binary, trans, and migrant women and PWM. Adequate facilities for menstrual management in public spaces, workplaces and educational centers are also required to address menstrual inequities. Besides, menstrual workplace policies can greatly aid menstrual management among working women and PWM. Overall, menstrual inequity policies must consider and be adapted to the needs of most affected populations, such as those more socioeconomically deprived, non-binary and trans PWM and vulnerabilised migrant populations.

## Conclusions

Our study presents an overview of menstrual inequities in Spain, which are widespread and affect a high number of women and PWM, especially those with a lower socioeconomic status, some migrant populations and non-binary and trans PWM. Working women and PWM also face increased challenges, particularly to manage menstruation in workplaces. Those with a higher educational attainment and socioeconomic status may have increased resources that serve as protective factors for menstrual inequities. Given that the study mostly assessed lifetime experiences of menstrual inequities, older participants seemed to be marginally more at risk for these social inequities. To our knowledge, this study presents the first data describing menstrual inequities in Spain. Its findings can have a great social impact and translate into policy, future research, practice and advocacy recommendations to tackle menstrual inequities. Very importantly, this study could aid addressing menstruation-related social inequities (of health).

## Supplementary Information

**Additional file 1: Supplementary Table 1.** Comparative between Equity and Menstrual Health in Spain Study Data (*N*=22,823) and Spanish National Statistics Institute Data.

**Additional file 2: Supplementary Table 2.** Access to healthcare services stratified by sociodemographic characteristics (N=22,313). **Supplementary** **Table 3.** Access to menstrual education and knowledge stratified by sociodemographic characteristics (N=21839). **Supplementary** **Table 4.** Menstrual management and menstrual poverty stratified by sociodemographic characteristics. **Supplementary** **Table 5.** Tabboo, embarrassement and experiences of stigma and discrimination stratified by sociodemographic characteristics. **Supplementary** **Table 6a.** Barriers to social, community and economic participation stratified by sociodemographic characteristics. **Supplementary** **Table 6b.** Barriers to social, community and economic participation stratified by sociodemographic characteristics.

## Data Availability

The datasets generated and analysed during the current study are not publicly available to maintain participants’ anonymity and confidentiality but are available from the corresponding author on reasonable request. The materials used for this study can also be accessed by contacting the corresponding author.

## Abbreviations

PWM: People who menstruate
