# Supplementary material for: Menstruation and social inequities in Spain: a cross-sectional online survey-based study
Source: Int J Equity Health. 2023 May 17;22:92. doi: 10.1186/s12939-023-01904-8 (PMC10189710; doi:10.1186/s12939-023-01904-8)
Supplement: Supplementary file 1 — Additional file 1: Supplementary Table 1. Comparative between Equity and Menstrual Health in Spain Study Data (N=22,823) and Spanish National Statistics Institute Data. [file 12939_2023_1904_MOESM1_ESM.docx]

**Supplementary Table 1. Comparative between Equity and Menstrual Health in Spain Study Data (N=22,823) and Spanish National Statistics Institute Data.**

|  | **Equity and Menstrual Health in Spain Study Data**  **(N=22,823)** | | **Spanish National Statistical Institute Data** |
| --- | --- | --- | --- |
| **Variable** | **N** | **%** | **%** |
| **Age (18-55)**  18-25  26-35  36-45  46-55 | *M=*33.2 (*SD=*8.7) | 24.0%  35.4%  31.8%  8.8% | 15.5%  22.5%  30.7%  31.3% |
| **Gender identification** Women  Non-binary/Other | 22,100 723 | 96.8% 3.2% | * |
| **Trans** Yes  Don’t know  No | 175 155  22493 | 0.8% 0.7%  98.6% | * |
| **Place of birth** Spain Not Spain | 20,943 1468 | 93.4% 6.6% | 76.4%  23.6% |
| **Administrative situation**  Spanish nationality No nationality | 21,785 895 | 95.5% 4.5% | 84.4%  15.6% |
| **Employment situation** Working full-time  Working part-time  Self-employed Studying full-time  Studying part-time  Unemployment /COVID-19 benefits  Pension or retirement Unpaid carer/houseworker | 10,834 3,914  2,050 3,896 1,934  1,831  163 1,134 | 47.5% 17.1%  9.0% 17.1% 8.5%  8.0%  0.7% 5.0% | 45.4%  10.1%  6.3%  25.6%  *  22.2%  1.6%  * |
| **Completed education** University education Secondary education Primary education No formal education completed | 15,790 6,665 250 35 | 69.2% 29.2% 0.9% 0.2% | 43.6%  51.5%  3.5%  1.4% |
| **Caregiving for someone else** Yes  No | 7,518  15,183 | 33.1%.  66.9% | * |
| **Financial problems in the last year** Always/many times Sometimes/a few times Never | 2,707 7,056 12,582 | 11.9% 30.9% 55.1% | ** |

Note. These data have been extracted from the Spanish National Statistics Institute ([www.ine.es](http://www.ine.es)) and correspond to representative data of women between 18 and 55 years old living in Spain in 2020.

*There are no representative data for these variables available.

**There are no representative data for this variable. However, the representative data available in Spain in 2018 revealed that 51.9% of women (aged 18-55) in Spain reported a high financial satisfaction, followed by 35.5% and 16.5% reporting moderate and low financial satisfaction respectively. Besides, 21.7% of women aged 18-55 in 2020 were at risk for relative poverty in Spain.
