# Supplementary material for: Menstruation and social inequities in Spain: a cross-sectional online survey-based study
Source: Int J Equity Health. 2023 May 17;22:92. doi: 10.1186/s12939-023-01904-8 (PMC10189710; doi:10.1186/s12939-023-01904-8)
Supplement: Supplementary file 2 — Additional file 2: Supplementary Table 2. Access to healthcare services stratified by sociodemographic characteristics (N=22,313). Supplementary Table 3. Access to menstrual education and knowledge stratified by sociodemographic characteristics (N=21839). Supplementary Table 4. Menstrual management and menstrual poverty stratified by sociodemographic characteristics. Supplementary Table 5. Tabboo, embarrassement and experiences of stigma and discrimination stratified by sociodemographic characteristics. Supplementary Table 6a. Barriers to social, community and economic participation stratified by sociodemographic characteristics. Supplementary Table 6b. Barriers to social, community and economic participation stratified by sociodemographic characteristics. [file 12939_2023_1904_MOESM2_ESM.docx]

**Supplementary Table 2. Access to healthcare services stratified by sociodemographic characteristics (N=22,313).**

|  | **Have accessed healthcare services for menstruation** | **Have not accessed but would like to** | **Have not accessed and do not think it is necessary** |
| --- | --- | --- | --- |
|  | N (%) | N (%) | N (%) |
| **Age**  18-25  26-35  36-45  46-55 | P<0.001  2821 (52.4%)  5065 (64.1%)  4613 (65.1%)  1313 (67.7%) | 1808 (33.6%)  1746 (22.1%)  1270 (17.9%)  259 (13.4%) | 756 (14.0%)  1094 (13.8%)  1201 (17.0%)  367 (18.9%) |
| **Gender identity**  Woman  Non-binary/Other | P=0.083  13404 (62.0%)  408 (59.3%) | 4929 (22.8%)  154 (22.4%) | 3292 (15.2%)  123 (18.3%) |
| **Trans**  Yes  I don’t know  No | P=0.029  86 (51.9%)  78 (62.0%)  13648 (55.7%) | 46 (27.5%)  35 (22.7%)  5002 (25.0%) | 35 (21.0%)  3356 (15.3%)  27 (19.3%) |
| **Country of birth**  Spain  Latin America  Europe  Other | P=0.004  12735 (62.2%)  476 (57.6%)  281 (57.2%)  66 (55.9%) | 4649 (22.7%)  207 (25.0%)  111 (22.6%)  33 (28.0%) | 3087 (15.1%)  144 (17.4%)  99 (20.2%)  19 (16.1%) |
| **Administrative situation**  Spanish nationality  Permanent residence  Temporal residence  No permit or in process | P<0.001  13274 (62.2%)  386 (54.5%)  95 (54.9)  24 (49.0%) | 4835 (22.7%)  169 (23.9%)  49 (28.3%)  16 (32.7%) | 3215 (15.1%)  153 (21.5%)  29 (16.8%)  9 (18.4%) |
| **Completed education**  Primary education  Secondary education  University education | P<0.001  136 (56.7%)  3720 (56.6%)  9945 (64.2%) | 59 (24.6%)  1818 (27.7%)  3199 (20.7%) | 45 (18.8%)  1030 (15.7%)  2337 (15.1%) |
| **Employment situation**  Working full-time/part-time  Self-employed  Studying full-time/part-time  Unemployment, COVID19 benefits**,** retirement and other benefits  Unpaid carer/houseworker | 9181 (63.9%) (p<0.001)  1353 (67.8%)(p<0.001)  3188 (55.9%) (p<0.001)  1218 (62.5%) (p=0.052)  705 (63.7%) (P=0.110) | 2935 (20.4%)  355 (17.8%)  1758 (30.8%)  468 (24.0%)  256 (23.1%) | 2260 (15.78%)  288 (14.4%)  760 (13.3%)  264 (13.5%)  145 (13.1%) |
| **Financial problems <12 months**  Always/Many times  Some/A few times  Never | P<0.001  1713 (64.5%)  4413 (63.8%)  7456 (60.6%) | 687 (25.69%)  1685 (24.4%)  2571 (20.9%) | 257 (9.7%)  818 (11.8%)  2273 (18.5%) |

*P<0.001

**Supplementary Table 3. Access to menstrual education and knowledge stratified by sociodemographic characteristics (N=21,839).**

|  | **Did not know what menstruation and the menstrual cycle were pre-menarche** | **Would like to have more information on menstruation and menstrual cycle** | **I don’t want more information, it’s not a topic that I’m interested in** | **Feeling intense menstrual pain is normal** |
| --- | --- | --- | --- | --- |
| **Age**  18-25  26-35  36-45  46-55 | P<0.001  580 (11.0%)  969 (12.5%)  933 (13.4%)  284 (14.86%) | P<0.001  4442 (87.4%)  6156 (82.2%)  4671 (70.3%)  1032 (56.5%) | 139 (2.7%)  197 (2.6%)  260 (3.9%)  84 (4.6%) | P<0.001  877 (16.4%)  973 (12.3%)  884 (12.4%)  210 (10.7%) |
| **Gender identity**  Woman  Non-binary/Other | P=0.177  2688 (12.7%)  78 (11.6%) | P=0.001  15813 (77%5  488 (75.2%) | 642 (3.1%)  38 (594%) | P=0.133  2838 (13.1%)  106 (15.0%) |
| **Trans**  Yes  I don’t know  No | P=0.141  23 (13.7%)  18 (13.3%) 2725 (12.7%) | P<0.001  106 (67.5%)  101 (77.7%)  16094 (77.7%) | 20 (12.7%)  7 (5.4%)  653 (3.1%) | P=0.003  34 (19.5%)  29 (19.3%)  2881 (13.1%) |
| **Country of birth**  Spain  Latin America  Europe  Other | P=0.001  2538 (12.7%)  100 (12.5%  63 (13.2%)  24 (20.2) | P=0.122  14995 (77.6%)  622 (80.4%)  340 (75.4%)  81 (75.7%) | 606 (3.1%)  30 (3.9%)  20 (4.4%)  3 (2.8%) | P=0.019  2676 (13.0%)  123 (15.0%)  78 (15.9%  24 (19.4%) |
| **Administrative situation**  Spanish nationality  Permanent residence  Temporal residence  No permit or in process | P=0.026  2636 (12.5%)  97 (14.2%)  20 (12.1%)  10 (21.7%) | P=0.212  15607 (77.5%)  490 (76.4%)  129 (80.6%)  41 (93.2%) | 649 (3.2%)  21 (3.3%)  6 (3.8%)  1 (2.3%) | P=0.064  2794 (13.1%)  105 (14.9%)  32 (19.0%)  6 (13.0%) |
| **Completed education**  Primary education  Secondary education  University education | P=0.104  36 (14.9%)  783 (12.2%)  1942 (12.8%) | P<0.001  153 (68.0%)  4871 (79.3%)  11263 (76.8%) | 10 (4.4%)  224 (3.6%)  444 (3.0%) | P<0.001  50 (20.3%  1068 (16.3%)  1822 (11.7%) |
| **Employment situation**  Working full-time/part-time  Self-employed  Studying full-time/part-time  Unemployment,COVID19 benefits**,** retirement and other benefits  Unpaid carer/houseworker | 1752 (12.5%) (p=0.346)  263 (13.4%) (p=0.073)  650 (11.7%) (p<0.001)  250 (13.1%) (p=0.515)  158 (14.4%) (p=0.010) | 10161 (75.0%) (p<0.001)  1454 (77.1%) (p=0.029)  4695 (86.8%) (p<0.001)  1462 (79.7%) (p=0.030)  822 (77.7%) (p=0.646) | 194 (2.6%)  44 (2.3%)  129 (2.4%)  46 (2.5%))  29 (2.7%) | 1762 (12.2%) (p<0.001)  224 (11.1%) (p=0.005)  893 (15.7%) (p<0.001)  271 (13.8%) (p=0.352)  120 (10.8%) (p=0.015) |
| **Financial problems <12 months**  Always/Many times  Some/A few times  Never | P<0.001  371 (14.4%)  918 (13.6%)  1424 (11.8%) | P<0.001  2100 84.5(%)  5295 (81.3%)  8550 (73.6%) | 56 (2.3%)  194 (3.0%)  415 (3.6%) | P<0.001  418 (15.8%)  972 (14.1%)  1486 (12.0%) |

**Supplementary Table 4. Menstrual management and menstrual poverty stratified by sociodemographic characteristics.**

|  | **Financial problems to access menstrual products  (N=21344)** | | | **Not able to choose menstrual products due to financial constraints  (N=21305)** | | | **Use of menstrual products longer than recommended/no adequate facilities  (N=21498)** | | | **Use of menstrual products longer than recommended/no product available  (N=21414)** | | |
| --- | --- | --- | --- | --- | --- | --- | --- | --- | --- | --- | --- | --- |
|  | **Always/Many times** | **Some/A few times** | **Never** | **Always/Many times** | **Some/A few times** | **Never** | **Always/Many times** | **Some/A few times** | **Never** | **Always/Many times** | **Some/A few times** | **Never** |
| **Age**  18-25  26-35  36-45  46-55 | P<0.001  128 (2.5%)  235 (3.1%)  130 (1.9%)  30 (1.6%) | 932 (18.5%)  1661 (21.9%)  1309 (19.2%)  372 (19.5%) | 3979 (79.0%)  5684 (75.0%)  5381 (78.9%)  1503 (78.9%) | P<0.001  593 (11.8%)  830 (11.0%)  525 (7.7%)  127 (6.7%) | 1542 (30.6%)  2423 (32.1%)  1996 (29.3%)  574 (30.3%) | 2909 (57.7%)  4302 (56.9%)  4289 (63.0%)  1195 (63.0%) | P<0.001  2141 (41.7%)  2572 (33.8%)  1677 (24.5%)  325 (17.1%) | 2749 (53.5%)  4664 (61.3%)  4732 (69.1%)  1409 (74.0%) | 245 (4.8%)  378 (5.0%)  436 (6.4%)  170 (8.9%) | P<0.001  1256 (24.5%)  1353 (17.9%)  692 (10.2%)  132 (7.0%) | 2985 (58.3%)  4576 (60.4%)  4060 (59.6%)  1055 (55.6%) | 879 (17.2%)  1650 (21.8%)  2064 (30.3%)  712 (35.7%) |
| **Gender identity**  Woman  Non-binary/Other | P<0.001  475 (2.3%)  481 (7.4%) | 4091 (19.8%)  183 (28.0%) | 16125 (77.9%)  422 (64.6%) | P<0.001  1964 (9.5%)  111 (17.1%) | 6291 (30.5%)  244 (37.5%) | 12400 (60.0%)  295 (45.4%) | P<0.001  6451 (31.0%)  264 (39.6%) | 13190 (63.3%)  364 (54.7%) | 1191 (5.7%)  38 (5.7%) | P<0.001  3276 (15.8%)  157 (23.5%) | 12283 (59.2%)  393 (58.9%) | 5188 (25.0%)  117 (17.5%) |
| **Trans**  Yes  No  I don’t know | P<0.001  8 (4.8%)  507 (2.4%)  8 (6.1%) | 51 (30.5%)  4179 (19.9%)  44 (33.6%) | 108 (64.7%)  16360 (77.7%)  79 (60.3%) | P<0.001  31 (18.7%)  2020 (9.6%)  24 (18.5%) | 64 (38.6%)  6422 (30.6%)  49 (37.7%) | 71 (42.8%)  12567 (59.8%)  57 (43.8%) | P<0.001  84 (49.1%)  6582 (31.1%)  49 (37.1%) | 83 (48.5%)  13393 (63.2%)  78 (59.1%) | 4 (2.3%)  1220 (5.8%)  5 (3.8%) | P<0.001  52 (30.2%)  3352 (15.9%)  29 (21.8%) | 97 (56.4%)  12497 (59.2%)  82 (61.7%) | 23 (13.4%)  5260 (24.9%)  22 (16.5%) |
| **Country of birth**  Spain  Latin America  Europe  Other | P<0.001  462 (2.4%)  38 (4.9%)  15 (3.2%)  2 (1.8%) | 3766 (19.2%)  273 (34.9%)  120 (25.6%)  48 (42.1%) | 15367 (78.4%)  472 (60.3%)  334 (71.2%)  64 (56.1%) | P<0.001  1875 (9.6%)  112 (14.4%)  47 (10.0%)  16 (13.8%) | 5886 (30.1%)  310 (39.9+%)  170 (36.3%)  48 (41.4%) | 11801 (60.3%)  355 (45.7%)  251 (53.6%)  52 (44.8%) | P<0.001  6122 (31.0%)  291 (37.1%)  138 (29.1%)  41 (35.0%) | 12518 (63.4%)  430 (54.8%)  310 (65.4%)  67 (57.3%) | 1097 (5.6%)  63 (8.0%)  26 (5.5%)  9 (7.7%) | P=0.017  3139 (16.0%)  152 (19.5%)  61 (13.0%)  25 (21.6%) | 11655 (59.3%)  424 (54.4%)  290 (61.8%)  64 (55.2%) | 4869 (24.8%)  204 (26.2%)  118 (25.2%)  27 (23.3%) |
| **Administrative situation**  Spanish nationality  Permanent residence  Temporal residence  No permit or in process | P<0.001  482 (2.4%)  27 (4.0%)  8 (4.9%)  2 (4.58%) | 3989 (19.5%)  191 (28.3%)  55 (33.7%)  31 (70.5%) | 15939 (78.1%)  458 (67.8%)  100 (61.3%)  11 (25.0%) | P<0.001  1943 (9.5%)  81 (12.1%)  28 (17.3%)  14 (31.1%) | 6172 (30.3%)  260 (38.8%)  68 (42.0%)  22 (48.9%) | 12260 (60.2%)  329 (49.1%)  66 (40.7%)  9 (20.0%) | P=0.020  6391 (31.1%)  227 (33.5%)  61 (37.4%)  19 (42.2%) | 13004 (63.2%)  408 (60.3%)  91 (55.8%)  20 (44.4%) | 1166 (5.7%)  42 (6.2%)  11 (6.7%)  6 (13.3%) | P=0.570  3276 (16.0%)  109 (16.3%)  34 (20.9%)  8 (18.2%) | 12128 (59.2%)  396 (59.1%)  98 (60.1%)  25 (56.8%) | 5081 (24.8%)  165 (24.6%)  31 (19.0%)  11 (25.0%) |
| **Completed education**  Primary education  Secondary education  University education | P<0.001  14 (6.3%)  257 (4.1%)  251 (1.7%) | 72 (32.3%)  1607 (25.9%)  2584 (17.4%) | 137 (61.4%)  4333 (69.9%)  12067 (81.0%) | P<0.001  36 (16.3%)  897 (14.5%)  1138 (7.7%) | 83 (37.6%)  2128 (34.3%)  4319 (29.1%) | 102 (46.2%)  3182 (51.3%)  9399 (63.3%) | P<0.001  62 (27.7%)  2204 (35.0%)  4444 (29.7%) | 140 (62.5%)  3744 (59.5%)  9659 (64.5%) | 22 (9.8%)  3241 (5.4%)  862 (5.8%) | P<0.001  34 (15.3%)  1239 (19.8%)  2158 (14.5%) | 120 (54.1%)  3691 (59.0%)  8852 (59.3%) | 68 (30.6%)  1325 (21.2%)  3906 (26.2%) |
| **Employment situation**  Working full-time/part-time  Self-employed  Studying full-time/part-time  Unemployment,COVID19 benefits**,** retirement and other benefits)  Unpaid carer/houseworker | 271 (2.0%) (p<0.001)  41 (2.1%) (p=0.635)  134 (2.5%) (p=0.613)  115 (6.1%) (p<0.001)  50 (4.6%) (p<0.001) | 2562 (18.6%)  387 (2.1%)  1105 (20.5%)  591 (31.4%)  329 (30.5%) | 10961 (79.5%)  1497 (77.8%)  4158 (77.0%)  1179 (62.5%)  698 (64.8%) | 1149 (8.4%) (p<0.001)  155 (8.0%) (p=0.019)  613 (11.3%) (p<0.001)  309 (16.4%) (p<0.001)  183 (17.0%) (p<0.001) | 4094 (29.8%)  583 (30.3%)  1752 (32.4%)  708 (37.5%)  385 (35.7%) | 8508 (61.9%)  1189 (61.7%)  3037 (56.2%)  869 (46.1%)  509 (47.3%) | 4067 (29.4%) (p<0.001)  506 (26.2%) (p<0.001)  2183 (39.7%) (p<0.001)  618 (32.6%) (p=0.382)  371 (34.2%) (p=0.049) | 9041 (65.2%)  1294 (67.0%)  3034 (55.2%)  1168 (61.6%)  646 (59.5%) | 748 (5.4%)  132 (6.8%)  277 (5.0%)  109 (5.8%)  68 (6.3%) | 2024 (14.7%) (p<0.001)  244 (12.7%) (p<0.001)  1230 (22.5%) (p<0.0001)  321 (17.0%) (p=0.072)  179 (16.6%) (p=0.881) | 8240 (59.7%)  1123 (58.4%)  3196 (58.5%)  1136 (60.3%)  636 (58.9%) | 3539 (25.6%)  557 (29.0%)  1041 (19.0%)  428 (22.7%)  265 (24.5%) |
| **Financial problems <12 months**  Always/Many times  Some/A few times  Never | P<0.001  329 (13.1%)  140 (2.1%)  49 (0.4%) | 1246 (49.6%)  1956 (29.9%)  1008 (8.5%) | 939 (37.4%)  4456 (68.0%)  10822 (91.1%) | P<0.001  749 (29.8%)  819 (12.5%)  464 (3.9%) | 1077 42.9(%)  2642 (40.3%)  2688 (22.7%) | 685 (27.3%)  3098 (47.2%)  8673 (73.3%) | P<0.001  1187 (46.7)  2305 (34.8%)  3086 (26.0%) | 12162 (49.7%)  4000 (60.3%)  8014 (67.5%) | 92 (3.6%)  327 (4.9%)  780 (6.6%) | P<0.001  701 (27.7%)  1149 (17.4%)  1499 (12.6%) | 1395 (55.2%)  3980 (60.4%)  7039 (59.4%) | 433 (17.1%)  1465 (22.2%)  3312 (27.9%) |

*Pvalue<0.001

**Supplementary Table 5. Tabboo, embarrassement and experiences of stigma and discrimination stratified by sociodemographic characteristics.**

|  | **Ashamed to buy or ask for menstrual products (N=21483)** | | | **Fear of staining in public (N=21045)** | | | **Frequency concealed menstruation  (N=20778)** | | | **Ashamed to talk about menstruation  (N=21004)** | | | **Ever felt discriminated or judged bc of menstruating (N=20406)** | | |
| --- | --- | --- | --- | --- | --- | --- | --- | --- | --- | --- | --- | --- | --- | --- | --- |
|  | **Always/Many times** | **Some/A few times** | **Never** | **Always/Many times** | **Some/A few times** | **Never** | **Always/Many times** | **Some/A few times** | **Never** | **Always/Many times** | **Some/A few times** | **Never** | **Always/Many times** | **Some/A few times** | **Never** |
| **Age**  18-25  26-35  36-45  46-55 | P<0.001  108 (2.1%)  67 (0.9%)  30 (0.4%)  8 (0.4%) | 922 (18.01%)  851 (11.2%)  605 (88%)  128 (6.7%) | 4069 (79.8%)  6702 (88.0%)  6224 (90.7%)  1769 (92.9%) | P<0.001  2523 (50.2%)  2471 (33.1%)  1957 (29.2%)  559 (30.1%) | 2018 (40.1%)  3714 (49.8%)  3399 (50.7%)  942 (50.7%) | 487 (9.7%)  1274 (17.1%)  1344 (20.1%)  357 (19.2%) | P<0.001  494 (9.9%)  694 (9.4%)  719 (10.9%)  219 (12.0%) | 2663 (53.4%)  3704 (50.3%)  3072 (46.6%)  774 (42.3%) | 1828 (36.7%)  2969 (40.3%)  2805 (42.5%)  837 (45.7%) | P<0.001  86 (1.7%)  86 (1.2%)  77 (1.2%)  14 (0.8%) | 1100 (21.9%)  1433 (19.2%)  1253 (18.8%)  316 (17.0%) | 3835 (76.4%)  5929 (79.6%)  5348 (80.1%)  1527 (82.2%) | P<0.001  335 (6.9%)  524 (7.2%)  314 (4.8%)  56 (3.1%) | 2017 (41.5%)  2946 (40.7%)  2302 (35.4%)  481 (26.6%) | 2503 (51.6%)  3772 (52.1%)  3885 (59.8%)  1271 (70.3%) |
| **Gender identity**  Woman  Non-binary/Other | P<0.001  177 (0.8%)  36 (5.5%) | 2400 (11.5%)  106 (16.2%) | 18250 (87.6%)  514 (78.4%) | P=0.003  7240 (35.5%)  270 (41.7%) | 9801 (48.1%)  272 (42.0%) | 3356 (16.5%)  106 (16.4%) | P<0.001  2022 (10.0%)  104 (16.5%) | 9889 (49.1%)  324 (51.5%) | 8238 (40.9%)  201 (32.0%) | P<0.001  240 (1.2%)  23 (3.6%) | 3943 (19.4%)  159 (24.7%) | 16176 (79.5%)  463 (71.8%) | P<0.001  1135 (5.7%)  94 (15.4%) | 7451 (37.6%)  295 (48.2%) | 11208 (56.6%)  223 (36.4%) |
| **Trans**  Yes  No  I don’t know | P<0.001  24 (14.5%)  186 (0.9%)  3 (2.3%) | 37 (22.3%)  2448 (11.6%)  21 (16.4%) | 105 (63.3%)  18555 (87.6%)  104 (81.3%) | P=0.004  79 (47.9%)  7374 (35.5%)  57 (44.2%) | 66 (40.0%)  9953 (48.0%)  54 (41.9%) | 20 (12.1%)  3424 (16.5%)  18 (14.0%) | P<0.001  35 (22.3%)  2069 (10.1%)  22 (17.6%) | 82 (52.2%)  10071 (49.1%)  60 (48.0%) | 40 (25.5%)  8356 (40.8%)  43 (34.4%) | P<0.001  12 (7.4%)  245 (1.2%)  6 (4.7%) | 56 (34.4%)  4014 (19.4%)  32 (24.8%) | 95 (58.3%)  16453 (79.4%)  91 (70.5%) | P<0.001  29 (19.1%)  1185 (5.9%)  15 (12.3%) | 71 (46.7%)  7609 (37.8%)  66 (54.1%) | 52 (34.2%)  11338 (56.3%)  41 (33.6%) |
| **Country of birth**  Spain  Latin America  Europe  Other | P<0.001  185 (0.9%)  10 (1.3%)  9 (1.9%)  4 (3.5%) | 2229 (11.3%)  131 (16.8%)  79 (16.7%)  29 (25.4%) | 17317 (97.8%)  639 (81.9%)  386 (81.4%)  81 (71.1%) | P=0.001  6874 (35.5%)  319 (41.9%)  144 (31.3%)  52 (46.4%) | 9286 (48.0%)  327 (43.0%)  241 (52.4%)  44 (39.3%) | 3178 (16.4%)  115 (15.1%)  75 (16.3%)  16 (14.3%) | P<0.001  1895 (9.9%)  118 (15.7%)  53 (11.8%)  19 (17.3%) | 9411 (49.3%)  352 (46.8%)  223 (49.4%)  54 (49.1%) | 7795 (40.8%)  282 (37.5%)  175 (38.8%)  37 (33.6%) | P<0.001  230 (1.2%)  15 (2.0%)  8 (1.7%)  6 (5.3%) | 3692 (19.1%)  175 (23.1%)  131 (28.6%)  36 (31.9%) | 15383 (79.7%)  566 (74.9%)  319 (69.7%)  71 (62.8%) | P=0.098  1113 (5.9%)  60 (8.1%)  27 (6.2%)  8 (7.5%) | 7128 (38.0%)  267 (35.8%)  179 (41.1%)  47 (43.9%) | 10511 (56.1%)  418 (56.1%)  229 (52.6%)  52 (48.6%) |
| **Administrative situation**  Spanish nationality  Permanent residence  Temporal residence  No permit or in process | P<0.001  192 (0.9%)  16 (2.4%)  3 (1.9%)  0 (0.0%) | 2336 (11.4%)  108 (16.0%)  40 (24.7%)  16 (37.2%) | 18023 (87.7%)  551 (81.6%)  119 (73.5%)  27 (62.8%) | P=0.003  7167 (35.6%)  238 (36.2%)  62 (40.0%)  28 (63.6%) | 9649 (47.9%)  307 (46.7%)  77 (49.7%)  13 (29.5%) | 3322 (16.5%)  113 (17.2%)  16 (10.3%)  3 (6.8%) | P<0.001  1999 (10.1%)  88 (13.7%)  25 (16.6%)  11 (25.0%) | 9797 (49.3%)  296 (46.0%)  80 (53.0%)  22 (50.0%) | 8084 (40.7%)  260 (40.4%)  46 (30.5%)  11 (25.0%) | P<0.001  245 (1.2%)  9 (1.4%)  6 (3.9%)  1 (2.3%) | 3865 (19.2%)  163 (24.9%)  46 (29.7%)  16 (36.4%) | 15992 (79.6%)  482 (73.7%)  103 (66.5%)  27 (61.4%) | P=0.280  1165 (6.0%)  42 (6.7%)  15 (10.1%)  4 (9.1%) | 7426 (38.0%)  230 (36.6%)  60 (40.3%)  14 (31.8%) | 10949 (56.0%)  336 (56.7%)  74 (49.7%)  26 (59.1%) |
| **Completed education**  Primary education  Secondary education  University education | P<0.001  1 (0.4%)  100 (1.6%)  112 (0.7%) | 16 (7.1%)  786 (12.6%)  1702 (11.4%) | 208 (92.4%)  5373 (85.8%)  13166 (87.9%) | P<0.001  98 (45.6%)  2716 (44.2%)  4692 (32.0%) | 77 (35.8%)  2550 (41.5%)  7435 (50.7%) | 40 (18.6%)  880 (14.3%)  2538 (17.3%) | P<0.001  27 (12.8%)  622 (10.2%)  1474 (10.2%) | 63 (29.9%)  2797 (46.0%)  7348 (50.8%) | 121 (57.3%)  2666 (43.8%)  5641 (39.0%) | P<0.001  5 (2.3%)  84 (1.4%)  174 (1.2%) | 28 (13.0%)  1048 (17.1%)  3022 (20.6%) | 182 (84.7%)  4998 (81.5%)  11445 (78.2%) | P<0.001  13 (6.3%)  423 (7.1%)  792 (5.6%) | 50 (24.0%)  2182 (36.7%)  5510 (38.7%) | 145 (69.7%)  3345 (56.2%)  7929 (55.7%) |
| **Employment situation**  Working full-time/part-time  Self-employed  Studying full-time/part-time  Unemployment,COVID19 benefits**,** retirement and other benefits)  Unpaid carer/houseworker | 97 (0.7%) (p<0.001)  15 (0.8%) (p=0.005)  94 (1.7%) (p<0.001)  22 (1.2%) (p=0.734)  11 (1.0%) (p=0.289) | 1431 (10.3%)  184 (9.5%)  903 (16.6%)  219 (11.5%)  110 (10.2%) | 12343 (89.0%)  1732 (89.7%)  4455 (81.7%)  1657 (87.3%)  961 (88.8%) | 4423 (32.6%) (p<0.001)  489 (25.8%) (p<0.001)  2464 (45.7%) (p<0.001)  702 (37.9%) (p=0.081)  367 (34.4%) (p=0.006) | 6798 (50.2%)  963 (50.8%)  2300 (42.6%)  844 (45.6%)  486 (45.6%) | 2334 (17.2%)  442 (23.3%)  631 (11.7%)  306 (16.5%)  213 (20.0%) | 1366 (10.2%) (p=0.852)  171 (9.2%) (p=0.001)  533 (10.0%) (p<0.001)  212 (11.6%) (p=0.120)  115 (10.9%) (p=0.003) | 6557 (49.0%)  863 (46.3%)  2815 (52.7%)  893 (48.9%)  464 (44.1%) | 5451 (40.8%)  829 (44.5%)  1994 (37.3%)  723 (39.6%)  474 (45.0%) | 156 (1.2%) (p=0.019)  21 (1.1%) (p=0.231)  81 (1.5%) (p<0.001)  29 (1.6%) (p=0.199)  13 (1.2%) (p=0.900) | 2583 (19.1%)  343 (18.2%)  1179 (21.9%)  340 (18.3%)  202 (19.0%) | 10787 (79.8%)  1525 (80.7%)  4119 (76.6%)  1484 (80.1%)  848 (79.8%) | 690 (5.2%) (p<0.001)  115 (6.2%) (p=0.599)  399 (7.7%) (p<0.001)  158 (8.8%) (p<0.001)  97 (9.4%) (p<0.001) | 4838 (36.7%)  716 (38.8%)  2230 (42.9%)  754 (42.1%)  440 (42.6%) | 7647 (58.0%)  1012 (54.9%)  2567 (49.4%)  879 (49.1%)  496 (48.0%) |
| **Financial problems <12 months**  Always/Many times  Some/A few times  Never | P<0.001  40 (1.6%)  77 (1.2%)  90 (0.8%) | 333 (13.1%)  814 (12.3%)  1275 (10.7%) | 2165 (85.3%)  5723 (86.5%)  10530 (88.5%) | P<0.001  1088 (43.9%)  2416 (37.2%)  3771 (32.4%) | 1022 (41.2%)  3106 (47.8%)  5788 (49.7%) | 370 (14.9%)  976 (15.0%)  2077 (17.8%) | P<0.001  3327 (13.7%)  646 (10.0%)  1096 (9.6%) | 1138 (46.4%)  3314 (51.5%)  5547 (48.4%) | 978 (39.9%)  2477 (38.5%)  4819 (42.0%) | P=0.065  40 (1.6%)  83 (1.3%)  135 (1.2%) | 495 (20.0%)  1311 (20.2%)  2201 (18.9%) | 1939 (78.4%)  5083 (78.5%)  9288 (79.9%) | P<0.001  318 (13.2%)  457 (7.3%)  431 (3.8%) | 1088 (45.2%)  2637 (41.9%)  3890 (34.4%) | 1001 (41.6%)  3202 (50.9%)  6982 (61.8%) |

**Supplementary Table 6a. Barriers to social, community and economic participation stratified by sociodemographic characteristics.**

|  | **Less capable to concentrate or deal with day-to-day activities when menstruating (N=20834)** | | | **Less productive when menstruating (N=18011)** | **Frequency not working when menstruating**  **(N=19123)** | | | **Frequency not studying when menstruating**  **(N=19057)** | | |
| --- | --- | --- | --- | --- | --- | --- | --- | --- | --- | --- |
|  | **Always/Many times** | **Some/A few times** | **Never** |  | **Always/Many times** | **Some/A few times** | **Never** | **Always/Many times** | **Some/A few times** | **Never** |
| **Age**  18-25  26-35  36-45  46-55 | P<0.001  1674 (33.6%)  1928 (26.1%)  1208 (18.2%)  215 (11.6%) | 2520 (50.7%)  3762 (51.0%)  3323 (50.2%)  794 (42.8%) | 781 (15.7%)  1692 (22.9%)  2093 (31.6%)  844 (45.5%) | P<0.001  4049 (90.1%)  5545 (84.8%)  4266 (77.3%)  916 (62.6%) | P<0.001  109 (3.2%)  200 (2.8%)  144 (2.2%)  24 (1.3%) | 694 (20.3%)  1328 (18.3%)  1078 (16.3%)  303 (16.5%) | 2624 (76.6%)  5723 (78.9%)  5388 (81.5%)  1508 (82.2%) | P<0.001  936 (19.0%)  970 (14.5%)  529 (9.12%)  78 (4.8%) | 2801 (56.8%)  35555 (53.8%)  2487 (42.8%)  584 (35.6%) | 1196 (24.2%)  2151 (32.2%)  2793 (48.1%)  977 (59.6%) |
| **Gender identity**  Woman  Non-binary/ Other | P<0.001  4780 (23.7%)  245 (38.5%) | 10111 (50.1%)  288 (45.3%) | 5307 (26.3%)  103 (16.2%) | P<0.001  14293 (81.9%)  483 (85.9%) | P<0.001  449 (2.4%)  28 (5.6%) | 3269 (17.5%)  134 (27.0%) | 14909 (80.0%)  334 (67.3%) | P<0.001  2381 (12.9%)  132 (21.9%) | 9104 (49.3%)  323 (53.6%) | 6969 (37.8%)  148 (24.5%) |
| **Trans**  Yes  I don’t know  No | P<0.001  72 (44.4%)  51 (40.5%)  4902 (23.9%) | 74 (45.7%)  57 (45.2%)  10268 (50.0%) | 16 (9.9%)  18 (14.3%)  5376 (26.2%) | P<0.001  137 (92.6%)  91 (85.0%)  14548 (81.9%) | P<0.001  5 (5.2%)  5 (5.3%)  467 (2.5%) | 28 (28.9%)  28 (29.5%)  3346 (29.5%) | 64 (66.0%)  62 (65.3%)  15117 (79.9%) | P<0.001  39 (25.2%)  23 (19.5%)  2451 (13.0%) | 85 (54.8%)  61 (51.7%)  9281 (49.4%) | 31 (20.0%)  34 (37.5%)  7052 (37.5%) |
| **Country of birth**  Spain  Latin America  Europe  Other | P=0.001  4593 (24.0%)  195 (25.8%)  128 (28.2%)  40 (36.0%) | 9558 (49.9%)  380 (50.2%)  239 (52.6%)  48 (43.2%) | 4989 (26.1%)  182 (24.0%)  87 (19.2%)  23 (20.7%) | P<0.001  13591 (82.1%)  543 (81.8%)  339 (84.9%)  79 (82.2%) | P<0.001  418 (2.4%)  31 (4.4%)  13 (3.1%)  3 (3.5%) | 3066 (17.5%)  165 (23.6%)  98 (23.1%)  23 (26.7%) | 14083 (80.2%)  502 (71.9%)  313 (73.8%)  60 (69.8%) | P=0.001  2295 (13.1%)  99 (14.30%)  57 (14.3%)  22 (21.69%) | 8718 (49.8%)  303 (42.7%)  204 (51.3%)  50 (49.0%) | 6495 (37.1%)  307 (43.3%)  137 (34.4%)  30 (29.4%) |
| **Administrative situation**  Spanish nationality  Permanent residence  Temporal residence  No permit or in process | P=0.001  4783 (24.0%)  161 (24.8%)  55 (35.9%)  17 (38.6%) | 9944 (49.9%)  337 (51.8%)  70 (45.8%)  21 (47.7%) | 5211 (26.1%)  152 (23.4%)  28 (18.3%)  6 (13.6%) | P<0.001  14129 (82.0%)  468 (84.2%)  119 (86.2%)  31 (73.8%) | P<0.001  446 (2.4%)  17 (2.8%)  6 (4.7%)  5 (12.8%) | 3199 (17.5%)  139 (22.7%)  40 (31.3%)  17 (43.6%) | 14662 (80.1%)  455 (74.5%)  82 (64.1%)  17 (43.6%) | P=0.264  2398 (13.1%)  76 (12.9%)  27 (17.9%)  5 (13.5%) | 9043 (49.6%)  271 (46.0%)  75 (49.7%)  20 (54.1%) | 6797 (37.3%)  242 (41.1%)  49 (32.5%)  12 (32.4%) |
| **Completed education**  Primary education  Secondary education  University education | P<0.001  49 (23.0%)  1722 (28.4%)  3248 (22.3%) | 91 (42.7%)  2896 (47.7%)  7406 (51.0%) | 73 (34.3%)  1453 (23.9%)  3879 (26.7%) | P<0.001  132 (74.2%)  4457 (84.4%)  10174 (81.1%) | P<0.001  9 (4.9%)  162 (3.4%)  305 (2.2%) | 34 (18.6%)  945 (19.6%)  2423 (17.2%) | 140 (76.5%)  3726 (77.1%)  11361 (80.6%) | P<0.001  16 (10.5%)  896 (16.1%)  1598 (12.0%) | 63 (41.2%)  2766 (49.8%)  6592 (49.4%) | 74 (48.4%)  1893 (34.1%)  5143 (38.6%) |
| **Employment situation**  Working full-time/part-time  Self-employed  Studying full-time/part-time  Unemployment,COVID19 benefits**,** retirement and other benefits)  Unpaid carer/houseworker | 2767 (20.6%) (p<0.001)  442 (23.5%) (p=0.781)  1747 (32.8%) (p<0.001)  533 (29.0%) (p<0.001)  286 (27.1%) (p=0.067) | 6703 (50.0%)  940 (50.0%)  2748 (51.5%)  903 (49.2%)  510 (48.3%) | 3949 (29.4%)  497 (26.5%)  838 (15.7%)  401 (21.8%)  260 (24.6%) | 9024 (79.1%) (p<0.001)  1351 (81.4%) (p=0.273)  4299 (89.4%) (p<0.001)  1383 (84.8%) (p=0.001)  755 (82.2%) (p=0.258) | 251 (1.9%) (p<0.001)  81 (4.3%) (p<0.001)  118 (3.1%) (p<0.001)  69 (4.2%) (p<0.001)  21 (2.3%) (p=0.015) | 2042 (15.1%)  503 (26.7%)  823 (21.7%)  318 (19.4%)  197 (21.3%) | 11253 (83.1%)  1298 (69.0%)  2849 (75.2%)  1256 (76.4%)  705 (76.4%) | 1308 (10.9%) (p<0.001)  218 (13.1%) (p=0.019)  1005 (18.7%) (p<0.001)  274 (16.2%) (p<0.001)  127 (13.7%) (p=0.899) | 5691 (47.2%)  771 (46.4%)  3135 (58.3%)  896 (53.0%)  457 (49.2%) | 5050 (41.9%)  671 (40.4%)  1241 (23.1%)  519 (30.7%)  344 (37.1%) |
| **Financial problems <12 months**  Always/Many times  Some/A few times  Never | P<0.001  921 (37.5%)  1812 (28.2%)  2188 (19.0%) | 1140 (46.5%)  3254 (50.6%)  5782 (50.2%) | 393 (16.0%)  1369 (21.3%)  3551 (30.8%) | P<0.001  1978 (88.5%)  4781 (85.3%)  7644 (78.6%) | P<0.001  111 (4.9%)  158 (2.7%)  199 (1.9%) | 556 (24.6%)  1251 (21.3%)  1549 (14.5%) | 1593 (70.5%)  4468 (76.0%)  8961 (83.7%) | P<0.001  498 (21.9%)  911 (15.3%)  1041 (10.0%) | 1187 (52.3%)  31678 (53.23%)  4869 (46.7%) | 586 (25.8%)  1873 (31.5%)  4515 (43.3%) |

*p=0.001

**Supplementary Table 6b. Barriers to social, community and economic participation stratified by sociodemographic characteristics.**

|  | **Frequency not exercising when menstruating**  **(N=19949)** | | | **Frequency not meeting friends/family when menstruating**  **(N=21027)** | | | **Frequency not going to the pool/beach when menstruating**  **(N=20609)** | | |
| --- | --- | --- | --- | --- | --- | --- | --- | --- | --- |
|  | **Always/Many times** | **Some/A few times** | **Never** | **Always/Many times** | **Some/A few times** | **Never** | **Always/Many times** | **Some/A few times** | **Never** |
| **Age**  18-25  26-35  36-45  46-55 | P<0.001  1957 (41.9%)  2442 (34.3%)  1695 (26.5%)  370 (21.1%) | 2157 (46.2%)  3706 (52.0%)  3526 (55.1%)  943 (53.7%) | 552 (11.8%)  978 (13.7%)  1179 (18.4%)  444 (25.3%) | P<0.001  448 (8.9%)  614 (8.2%)  412 (6.2%)  82 (4.4%) | 2663 (53.1%)  3696 (49.5%)  2819 (42.2%)  614 (33.0%) | 1908 (38.0%)  3151 (42.2%)  3454 (51.7%)  1165 (62.6%) | P<0.001  1612 (32.7%)  1703 (23.4%)  1465 (22.3%)  401 (21.9%) | 2298 (46.7%)  3618 (49.6%)  3282 (50.0%)  885 (48.4%) | 1015 (20.6%)  1968 (27.0%)  1818 (27.7%)  544 (29.7%) |
| **Gender identity**  Woman  Non-binary/ Other | P<0.001  6206 (32.0%)  25/8 (44.2%) | 10079 (52.0%)  253 (43.3%) | 3080 (15.9%)  73 (12.5%) | P<0.001  1473 (7.2%)  84 (13.0%) | 9437 (46.3%)  355 (55.1%) | 9473 (46.5%)  205 (31.8%) | P<0.001  4935 (24.7%)  246 (40.1%) | 9819 (49.1%)  264 (43.1%) | 5242 (26.2%)  103 (16.8%) |
| **Trans**  Yes  I don’t know  No | P<0.001  70 (50.7%)  45 (40.8%)  6349 (32.2%) | 57 (41.3%)  53 (47.7%)  10222 (51.9%) | 11 (8.0%)  13 (11.7%)  3129 (15.9%) | P<0.001  20 (12.2%)  13 (10.2%)  1524 (7.3%) | 95 (57.9%)  70 (54.7%)  9627 (46.4%) | 49 (29.9%)  45 (35.2%)  9584 (46.2%) | P<0.001  83 (56.5%)  49 (40.5%)  5049 (24.8%) | 51 (34.7%)  52 (43.0%)  9980 (49.1%) | 13 (8.8%)  20 (16.5%)  5312 (26.1%) |
| **Country of birth**  Spain  Latin America  Europe  Other | P=0.016  5902 (32.2%)  264 (36.7%)  162 (37.8%)  37 (34.9%) | 9525 (51.9%)  351 (48.8%)  213 (49.7%)  58 (54.7%) | 2909 (15.9%)  105 (14.6%)  54 (12.6%)  11 (10.4%) | P=0.002  1406 (7.3%)  81 (10.7%)  35 (7.7%)  11 (9.7%) | 9023 (46.7%)  332 (43.9%)  222 (48.7%)  64 (56.6%) | 8900 (46.0%)  344 (45.4%)  199 (43.6%)  38 (33.6%) | P<0.001  4645 (24.5%)  285 (39.1%)  142 (31.9%)  35 (33.0%) | 9317 (49.1%)  312 (42.8%)  212 (47.6%)  55 (51.9%) | 5004 (26.4%)  132 (18.1%)  91 (20.4%)  16 (15.1%) |
| **Administrative situation**  Spanish nationality  Permanent residence  Temporal residence  No permit or in process | P=0.105  6164 (32.3%)  208 (33.4%)  65 (43.0%)  14 (36.8%) | 9902 (51.9%)  324 (52.0%)  66 (43.7%)  21 (55.3%) | 3027 (15.9%)  91 (14.6%)  20 (13.2%)  3 (7.9%) | P=0.058  1477 (7.3%)  52 (7.9%)  17 (11.0%)  8 (19.0%) | 9375 (46.6%)  301 (46.0%)  72 (46.5%)  19 (42.5%) | 9274 (46.1%)  302 (46.1%)  66 (42.6%)  15 (37.5%) | P<0.001  4868 (24.7%)  216 (33.9%)  60 (40.5%)  25 (67.6%) | 9700 (49.1%)  292 (45.8%)  57 (38.5%)  10 (27.0%) | 5170 (26.2%)  130 (20.4%)  31 (20.9%)  2 (5.4%) |
| **Completed education**  Primary education  Secondary education  University education | P<0.001  53 (29.0%)  2053 (36.3%)  4355 (30.9%) | 88 (48.1%)  2698 (47.7%)  7533 (53.5%) | 42 (23.0%)  906 (16.0%)  2202 (15.6%) | P<0.001  18 (8.5%)  555 (9.1%)  982 (6.7%) | 81 (38.0%)  2989 (48.8%)  6711 (45.8%) | 114 (53.5%)  2584 (42.2%)  6975 (47.6%) | P<0.001  57 (27.7%)  1820 (30.5%)  3299 (22.9%) | 89 (43.2%)  2759 (46.2%)  7226 (50.1%) | 60 (29.1%)  1396 (23.4%)  3884 (27.0%) |
| **Employment situation**  Working full-time/part-time  Self-employed  Studying full-time/part-time  Unemployment,COVID19 benefits**,** retirement and other benefits  Unpaid carer/houseworker | 3821 (29.5%) (p<0.001)  563 (31.2%) (p=0.048)  2057 (41.0%) (p<0.001)  6314 (36.4%) (p<0.001)  345 (34.8%) (p=0.260) | 6924 (53.4%)  920 (51.0%)  2396 (47.7%)  881 (50.8%)  495 (49.9%) | 2227 (17.2%)  321 (17.8%)  570 (11.3%)  222 (12.8%)  152 (15.3%) | 861 6.4(%) (p<0.001)  150 (7.9%) (p=0.151)  492 (9.1%) (p<0.001)  206 (11.1%) (p<0.001)  97 (9.1%) (p=0.061) | 5967 (44.0%)  841 (44.5%)  2881 (53.5%)  950 (51.2%)  500 (47.0%) | 6725 (49.6%)  899 (47.6%)  2012 (37.4%)  700 (37.7%)  467 (43.9%) | 2887 (21.7%) (p<0.001)  412 (22.3%) (p<0.001)  1668 (31.7%) (p<0.001)  551 (30.6%) (p<0.001)  319 (30.6%) (p<0.001) | 6676 (50.1%)  891 (48.2%)  2531 (48.1%)  863 (47.9%)  486 (46.6%) | 3751 (28.2%)  547 (29.6%)  1066 (20.2%)  386 (21.4%)  237 (22.7%) |
| **Financial problems <12 months**  Always/Many times  Some/A few times  Never | P<0.001  1006 (43.8%)  2241 (36.3%)  3067 (27.7%) | 1013 (44.1%)  3122 (50.6%)  6003 (54.2%) | 279 (12.1%)  807 (13.1%)  2010 (18.1%) | P<0.001  366 (14.8%)  577 (8.9%)  587 (5.0%) | 1286 (52.0%)  3306 (51.0%)  4979 (42.8%) | 820 (33.2%)  2604 (40.1%)  6071 (52.2%) | P<0.001  873 (36.3%)  1777 (28.0%)  2385 (20.9%) | 1053 (43.8%)  3168 (49.8%)  5672 (49.6%) | 480 (20.0%)  1411 (22.2%)  3368 (29.5%) |
